# Current Advances in Cetacean Semen Cryopreservation and Their Application to Yangtze Finless Porpoise Conservation

**DOI:** 10.3390/ani16142191

**Published:** 2026-07-14

**Authors:** Qingyue Wang, Congping Ying, Chu Wang, Jialu Zhang, Danqing Lin, Kai Liu, Shengyan Su

**Affiliations:** 1 Key Laboratory of Freshwater Fisheries and Germplasm Resources Utilization, Ministry of Agriculture and Rural Affairs, Freshwater Fisheries Research Center, Chinese Academy of Fishery Sciences, Wuxi 214081, China; 15094386833@163.com (Q.W.); yingcongping@ffrc.cn (C.Y.); 19281843551@163.com (C.W.); zhangjialu@ffrc.cn (J.Z.); lindq@ffrc.cn (D.L.); 2 Wuxi Fisheries College, Nanjing Agricultural University, Wuxi 214081, China

**Keywords:** sperm cryopreservation, cetaceans, Yangtze finless porpoise, germplasm resource conservation, assisted reproduction, cryobiology, biodiversity conservation, reproductive biotechnology

## Abstract

Cryopreservation of semen is an important interdisciplinary field at the intersection of reproductive biotechnology and cryobiology, and holds significant value for the genetic rescue of endangered species, long-term preservation of germplasm resources, and management of reproduction in livestock and aquaculture. Although this technology is relatively well established in fish and terrestrial mammals, it still faces numerous challenges in marine mammals, particularly cetaceans. The Yangtze finless porpoise (*Neophocaena asiaeorientalis asiaeorientalis*), a rare and endemic freshwater porpoise of China, remains at an early stage of research regarding semen cryopreservation, and there is an urgent need to develop a technical system tailored to its unique physiological characteristics. Literature was searched in PubMed, Web of Science, and CNKI from database inception to May 2025. This structured narrative review was based on 77 core publications, including eight original studies on cetacean semen cryopreservation. This structured narrative review synthesizes evidence from cetacean semen cryopreservation research, with particular emphasis on technical insights from the bottlenose dolphin (*Tursiops truncatus*), Pacific white-sided dolphin (*Lagenorhynchus obliquidens*), killer whale (*Orcinus orca*), and beluga (*Delphinapterus leucas*). Additionally, it incorporates advances in extender formulations and cryoprotectants from studies on other species to summarize key technical parameters suitable for the cryopreservation of Yangtze finless porpoise semen, aiming to provide a theoretical basis and practical framework for establishing an efficient and safe cryopreservation system for this species.

## Simple Summary

Sperm cryopreservation is a technique that freezes and stores sperm at ultra-low temperatures, enabling long-term preservation of genetic material from endangered animals. While widely used in many land animals and fish, it remains difficult to apply to cetaceans such as dolphins, whales, and porpoises. Unlike terrestrial mammals, cetacean sperm have distinct structural features, including a flattened head, unique post-acrosomal structures, and higher seminal plasma osmolality, which influence their response to freezing and thawing. The Yangtze finless porpoise, a critically endangered freshwater porpoise found only in China’s Yangtze River, urgently needs such a method to protect its declining genetic diversity. Because each species has unique sperm characteristics, protocols developed for other species cannot be directly applied without species-specific optimization. This review brings together current knowledge from several cetacean species, including bottlenose dolphins, Pacific white-sided dolphins, killer whales, and belugas, and also examines advances from other animal studies. By integrating these cross-species insights, we aim to provide a scientific foundation for designing an optimized freezing protocol tailored to the Yangtze finless porpoise, which will support future artificial breeding and genetic resource banking for this unique and threatened species.

## 1. Introduction

Many cetaceans occupy high trophic levels and play important ecological roles in maintaining food web stability and ecological balance [1]. However, their highly specialized reproductive strategies, shaped by long-term evolution including seasonal breeding [2], complex mating systems [3,4,5], and low reproductive rates [2,6] make them highly sensitive to environmental disturbances [7,8]. At present, global cetacean populations face multiple anthropogenic threats, such as habitat fragmentation, overfishing, climate change [8], water pollution [9], and shipping noise [10], leading to increasingly severe conservation challenges. Consider, for example, the Yangtze finless porpoise, the only freshwater cetacean endemic to the Yangtze River basin in China. It is listed as Critically Endangered by the International Union for Conservation of Nature (IUCN) [11] and is strictly protected as a First Class National Protected Animal. Thanks to the ambitious “Great Protection of the Yangtze River” strategy and the 10-year fishing ban, its population has shown a recovery trend, increasing from approximately 1012 individuals in 2017 (based on visual surveys) to about 1426 in 2025 (based on visual surveys supplemented by acoustic monitoring). Nevertheless, its critically endangered status has not yet been fundamentally reversed. Population genomic studies have revealed extremely low genetic diversity, with an effective population size (Ne) of fewer than 92 individuals per population and observed heterozygosity (Ho) as low as 0.653 in some populations, indicating that its genetic diversity remains highly fragile [12,13,14].

Given the critical conservation status of cetaceans, the development of advanced assisted reproductive technologies, particularly semen cryopreservation, is recognized as a key strategy to overcome existing bottlenecks. This technology allows the long-term, stable preservation of a species’ genetic information, providing an important tool for long-term conservation of genetic diversity [15]. For cetaceans—characterized by low reproductive rates and long generation intervals semen cryopreservation is of particular value: it can help maintain and enhance genetic diversity in captive populations, establish genome banks to mitigate extinction risks, and supply valuable genetic resources for future population restoration. However, the successful application of this technology to freshwater cetaceans such as the Yangtze finless porpoise faces formidable challenges. The freeze–thaw process can damage spermatozoa through ice crystal formation, osmotic shock, oxidative stress, and disruption of membrane integrity, with the extent of damage varying across species [16]. Significant interspecific differences exist not only among different taxa but also among closely related genera, in terms of sperm plasma membrane lipid composition, aquaporin expression and function, membrane cholesterol/phospholipid ratio, mitochondrial physiology, intracellular osmoregulatory mechanisms, antioxidant defense capacity, and sensitivity to cryoprotective agents (CPAs) [17,18,19,20,21,22,23]. These factors, together with other cellular attributes, are increasingly recognized as critical determinants of cryotolerance, contributing to the highly species-specific response of spermatozoa to freeze–thaw stress [24]. Therefore, mature protocols developed for terrestrial mammals cannot be directly applied; systematic research targeting each species of interest is essential. Although preliminary progress has been made in marine cetaceans such as the bottlenose dolphin and the beluga, current technologies remain far from being stable, efficient, and widely applicable.

Nevertheless, freshwater cetaceans like the Yangtze finless porpoise still lack validated, species-specific technical systems, which constitutes a critical bottleneck limiting the improvement of conservation and genetic resource management. The objectives of this review were to: (1) summarize current knowledge on cetacean semen cryopreservation, (2) compare cryopreservation protocols across species, (3) identify methodological gaps, and (4) propose future research priorities for the conservation of the Yangtze finless porpoise. By achieving these objectives, this review aims to provide a theoretical basis and a practical framework for the development of an efficient and safe cryopreservation system tailored to the Yangtze finless porpoise, thereby supporting cetacean conservation and genetic resource management.

## 2. Literature Search Strategy and Review Methodology

The aim of this review was to synthesize current advances in cetacean semen cryopreservation, with particular emphasis on the conservation needs of the Yangtze finless porpoise (*Neophocaena asiaeorientalis asiaeorientalis*). Because the available literature is highly heterogeneous, including case reports, species-specific protocol studies, reproductive biology reports, and methodological references from other mammals, a quantitative meta-analysis was not feasible. Therefore, this review adopted a narrative review approach supported by a structured literature search. This strategy allowed a comprehensive and critical thematic synthesis while acknowledging the methodological diversity and limitations of the field.

### 2.1. Literature Search Platforms and Databases

Literature searches were conducted in PubMed, Web of Science, and China National Knowledge Infrastructure (CNKI) to cover both international peer-reviewed studies and Chinese-language literature. The search covered all records available from database inception to May 2025. Online early-view papers available by this date were also considered eligible, including relevant studies formally assigned to later publication years.

Google Scholar was not used as a primary search database because its ranking algorithm is not fully reproducible and because its coverage includes grey literature that was not used as the primary evidence base in this review. However, to reduce the risk of missing important theses, book chapters, and older foundational publications, backward and forward citation tracking was performed as a supplementary search strategy.

### 2.2. Search Terms and Search Strategy

Search terms were developed in two complementary categories.

For the core subject search on cetacean semen cryopreservation, the following English search string was used in PubMed and Web of Science:

(cetacean* OR dolphin* OR porpoise* OR whale* OR Neophocaena OR Tursiops OR Orcinus OR Delphinapterus OR Lagenorhynchus) AND (semen OR sperm* OR spermatozoa) AND (cryopreserv* OR cryobiology OR freezing OR cryostorage OR “freeze–thaw” OR vitrification OR “semen preservation” OR “gamete preservation” OR “sperm banking” OR “gene banking” OR “genome resource banking” OR “male fertility”)

For CNKI, the following Chinese search terms were used:

(鲸类 OR 海豚 OR 江豚 OR 虎鲸 OR 白鲸 OR 长江江豚) AND (精液 OR 精子) AND (冷冻保存 OR 超低温冷冻 OR 冻融 OR 低温保存)

A supplementary search focusing on Yangtze finless porpoise reproductive physiology was also conducted using:

(长江江豚) AND (生殖 OR 性腺 OR 睾丸 OR 精液)

To identify methodological references from terrestrial mammals and human assisted reproduction, an additional search was performed without species restriction using the following English terms:

(semen OR sperm) AND (cryopreserv OR freezing OR cryostorage OR semen preservation OR gamete preservation OR sperm banking) AND (antioxidant* OR extender OR diluent OR glycerol OR egg yolk OR trehalose OR density gradient centrifugation OR DNA fragmentation OR birefringence OR pentoxifylline)

Relevant journals, including Theriogenology, Animal Reproduction Science, Cryobiology, and Reproduction in Domestic Animals, were also searched for methodological studies. In CNKI, the supplementary methodological search used:

(精液 OR 精子) AND (冷冻保存) AND (抗氧化剂 OR 稀释液 OR 甘油 OR 卵黄 OR 密度梯度离心)

### 2.3. Literature Screening Process

Using the core subject search strategy described above, 41 unique records were identified from PubMed, Web of Science, and CNKI after deduplication. To capture additional methodologically relevant studies from terrestrial mammals and human assisted reproduction, a structured snowballing strategy was then applied. This included backward citation searching of the reference lists of core articles and major reviews, as well as forward citation searching using the “Times Cited” function in Web of Science to identify recent studies citing key publications. This process yielded an additional 36 records. Snowballing was terminated when three consecutive rounds of citation tracking failed to identify any new methodological variants or species-specific data relevant to the objectives of this review.

Together, the database search and snowballing process yielded 77 records for screening. After stepwise assessment against the eligibility criteria, 77 records were included in the core evidence base. These comprised 68 English journal articles, 3 Chinese journal articles, 3 Chinese dissertations 1 Chinese patent, and 2 English book chapters. By source of evidence, approximately 41 records were cetacean-related, whereas approximately 36 records were derived from terrestrial mammals and human assisted reproduction and were used as methodological references.

In addition to these 77 core records, 46 supplementary references were cited to provide essential biological, conservation, and technical context. These included studies on cetacean reproductive anatomy, endocrine regulation, sperm morphology, ecological threats, conservation status, and methodological innovations from related fields such as aquaculture, stem cell biology, and molecular dynamics simulation. These supplementary references were not subjected to the same eligibility criteria as the core evidence base, but were retained to support the biological and technical rationale of the review. Therefore, this review cites a total of 124 references.

The literature screening was conducted by a single reviewer (the corresponding author), which is an accepted approach for highly specialized narrative reviews but should be recognized as a methodological limitation. Because only one reviewer performed the screening, no inter-reviewer disagreement process was applicable. To improve consistency and reproducibility, all records were screened according to predefined inclusion and exclusion criteria (Section 2.4), and ambiguous records were re-evaluated after a 48 h interval before the final inclusion decision.

### 2.4. Inclusion and Exclusion Criteria

During title and abstract screening, records were excluded if they were not directly related to semen cryopreservation, sperm functional assessment, assisted reproduction, or relevant reproductive biology; if they were not written in English or Chinese; or if they were conference abstracts, editorial letters, or duplicates.

During full-text screening, studies were excluded if they reported only anatomical observations or basic semen parameters without relevance to cryopreservation, post-thaw sperm quality, or assisted reproduction. Studies lacking specific methodological information, such as cryoprotectant composition, freezing protocol, thawing procedure, or post-thaw assessment parameters, were also excluded from the core experimental evidence base.

Background literature on reproductive anatomy, endocrine profiles, sperm morphology, life history, and conservation status was retained separately and was not subjected to the same eligibility criteria as the core cryopreservation studies.

### 2.5. Data Extraction and Synthesis

Because the included studies differed substantially in species, semen collection methods, extender composition, cryoprotectant type and concentration, cooling rate, thawing procedure, and sperm quality assessment methods, meta-analysis was not appropriate. A narrative synthesis was therefore conducted.

Extracted information included species, semen collection method, extender composition, osmolality, cryoprotectant type and concentration, packaging format, cooling rate, thawing condition, post-thaw motility, viability, acrosome integrity, DNA fragmentation, and artificial insemination outcomes. For background studies, key information on reproductive anatomy, endocrine regulation, seasonality, and sperm morphology was summarized.

The review was organized thematically into reproductive physiology and semen characteristics, semen collection techniques, cryopreservation and thawing protocols, post-thaw sperm assessment, and applications in assisted reproduction. Evidence from bottlenose dolphins, Pacific white-sided dolphins, killer whales, and belugas was compared where available. Data from the Yangtze finless porpoise and related finless porpoise species were integrated to identify knowledge gaps and to inform future cryopreservation strategies.

### 2.6. Quality Assessment and Limitations

Because this was a narrative review, no formal quantitative risk-of-bias tool was applied. Instead, study quality was assessed qualitatively throughout the review, with attention to study design, sample size, use of controls, replication, protocol reproducibility, assessment endpoints, and whether samples originated from captive or wild animals. Several methodological limitations should be acknowledged. First, the literature on cetacean reproductive biology and semen cryopreservation remains scarce and is derived from relatively few research groups and zoological institutions, resulting in small sample sizes and an increased risk of publication bias. Second, screening was performed by a single reviewer without independent duplicate assessment, which may have introduced selection bias despite the use of predefined eligibility criteria and repeated screening of ambiguous records. Third, many methodological recommendations rely partly on evidence from terrestrial mammals and human assisted reproduction because cetacean-specific data remain limited. Finally, this review was not prospectively registered and did not employ a formal risk-of-bias assessment tool, reflecting its narrative rather than systematic review design. These limitations were considered when interpreting the available evidence and formulating future research priorities.

Throughout this review, evidence is interpreted according to a hierarchical framework. Direct evidence from the Yangtze finless porpoise is presented preferentially whenever available, followed by evidence from the East Asian finless porpoise, other cetacean species, and finally terrestrial mammals or human reproductive medicine when cetacean-specific data are lacking. Recommendations based on cross-species extrapolation are explicitly identified as hypotheses requiring species-specific validation.

## 3. Overview of Cetacean Reproductive Physiology and Semen Characteristics

### 3.1. Anatomical and Physiological Features of the Male Cetacean Reproductive System

**Figure 1:**
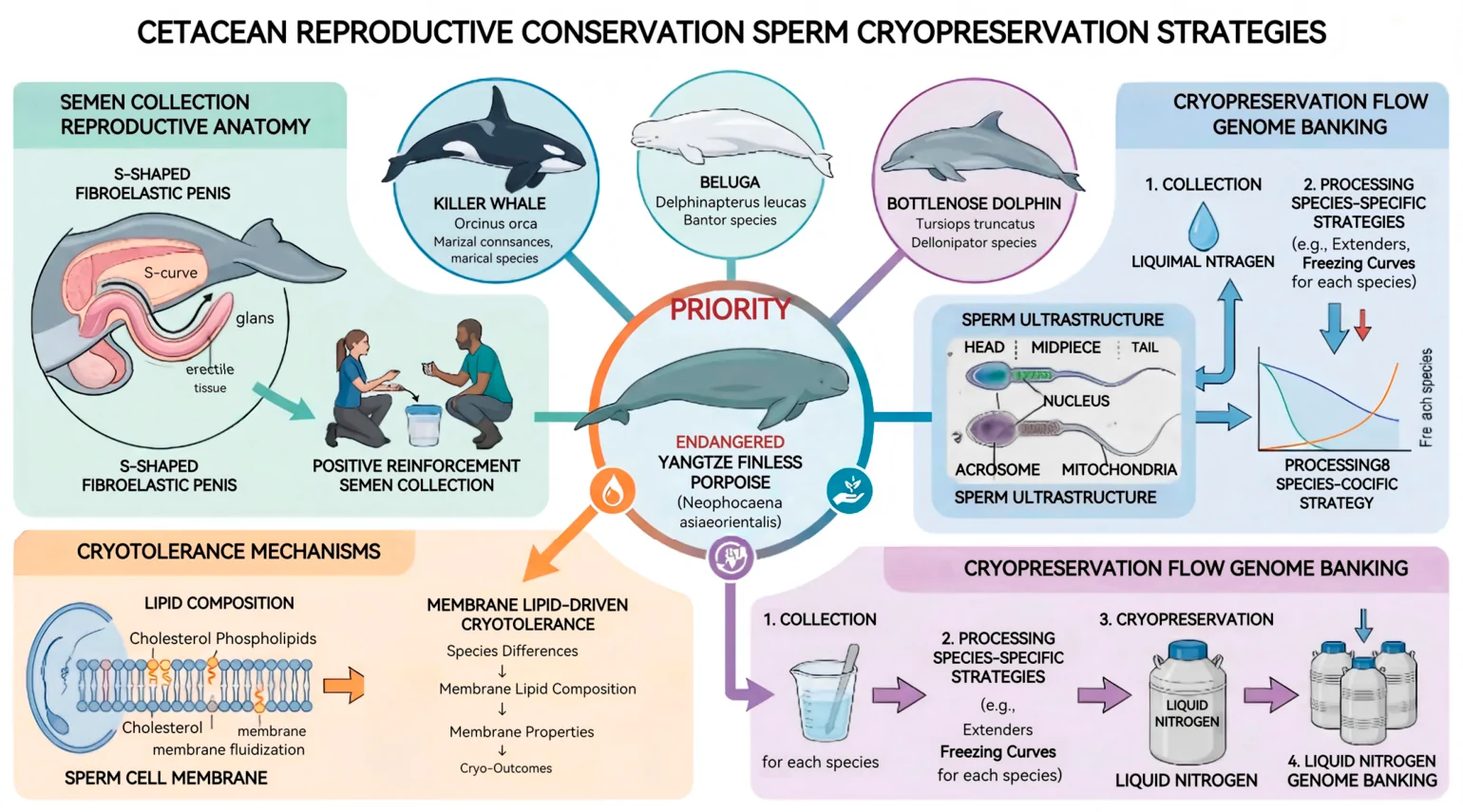
Integrated framework linking male reproductive anatomy, semen characteristics, cryopreservation challenges, and conservation strategies in cetaceans, highlighting the Yangtze finless porpoise as a priority conservation target.

As fully aquatic mammals, cetaceans have undergone significant morphological, physiological, and molecular remodeling of the male reproductive system during evolution to adapt to their unique aquatic environment and reproductive strategies. These specializations are key to understanding their reproductive biology and developing assisted reproductive technologies, such as semen cryopreservation. These anatomical and physiological adaptations are also expected to influence sperm membrane composition, osmotic regulation, and cryoresistance, highlighting the need for species-specific cryopreservation protocols (Figure 1).

The penis of marine cetaceans is of the fibroelastic type, which in the flaccid state is kept retracted within the body via a sigmoid flexure controlled by the retractor penis muscle [25]. During copulation, it rapidly extends and acquires sufficient rigidity for intromission in the aquatic environment [26]. From a reproductive management perspective, this anatomical configuration, particularly the sigmoid flexure and the fibroelastic nature of the penis, has direct implications for semen collection protocols, as successful artificial vagina or electroejaculation procedures must accommodate these structural features to minimise stress and injury to the animal.

**Figure 2:**
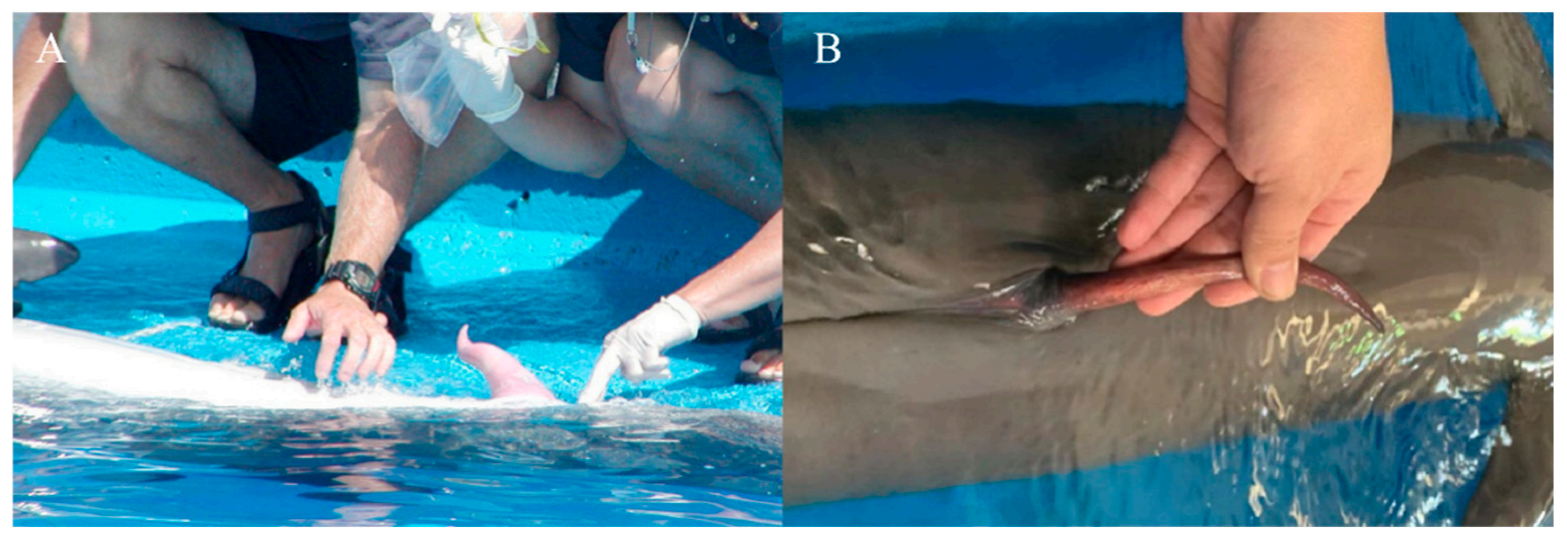
External genitalia of a male bottlenose dolphin (*T. truncatus*) [35] (**A**) and a male Yangtze finless porpoise (*N. asiaeorientalis asiaeorientalis*) (**B**). The anatomical features shown provide the basis for semen collection and the development of species-appropriate cryopreservation procedures.

In terms of reproductive physiology, the bottlenose dolphin (Tursiops truncatus) is one of the most thoroughly studied marine cetacean species (Figure 2A shows the reproductive organs of a bottlenose dolphin). An 8–12-year longitudinal study showed that inhibin levels were significantly higher before sexual maturity than after maturity, while follicle stimulating hormone (FSH) levels increased after maturity, with a negative correlation between the two, indicating that inhibin suppresses FSH secretion during the immature period [27]. Notably, the age of sexual maturity in captive male bottlenose dolphins (approximately 17 years) is later than that in wild populations (8–11 years), which may be related to differences in environment, nutrition, or social structure [28].

Furthermore, testosterone levels in bottlenose dolphins typically peak in spring (March–June), but the highest sperm concentration has been observed in autumn (October) [29]; this temporal mismatch between the hormone peak and the peak in sperm quality suggests the complexity of their reproductive regulation and has practical implications for scheduling semen collection to obtain optimal samples [28]. Several biological factors may account for this asynchrony. First, the duration of spermatogenesis in cetaceans is estimated to be approximately 7–8 weeks, which could introduce a delay between the hormonal signal initiating spermatogenesis and the production of mature spermatozoa. Second, epididymal maturation and storage represent an additional time lag before spermatozoa become fully functional and available for ejaculation. Third, seasonal endocrine regulation particularly the progressive decline of testosterone after its peak may create a physiological environment that favours the final maturation and release of spermatozoa, leading to a delayed peak in sperm output [28,30,31,32]. Understanding these temporal dynamics is relevant for cryopreservation, as spermatozoa collected at different phases of the seasonal cycle may exhibit differences in membrane composition and intrinsic cryotolerance.

Unlike the bottlenose dolphin, the killer whale exhibits a significant increase in testosterone levels from spring to early autumn, but sperm concentration does not show marked seasonal fluctuations, suggesting that male killer whales are reproductively competent throughout the year [33]. This apparent lack of marked reproductive seasonality has an important practical implication for conservation: it would theoretically facilitate year-round semen collection, which represents a significant logistical advantage for cryobanking programs by providing greater flexibility in sample acquisition and reducing the urgency associated with narrow seasonal windows. For the East Asian finless porpoise (*Neophocaena asiaeorientalis sunameri*), which is most closely related to the Yangtze finless porpoise, 3–3.5 years of age is a critical turning point in testicular development: seminiferous tubule diameter (STD), tunica albuginea thickness (TTA), and testis mass (TM) all increase markedly at this stage, and a small number of spermatozoa are detected for the first time, marking the onset of puberty; full sexual maturity (with abundant spermatozoa visible in the seminiferous tubules) is reached at approximately 5 years of age. Moreover, testosterone levels in the East Asian finless porpoise increase sharply after 3 years of age, whereas estradiol is highly expressed in immature individuals and declines with age. In addition, the androgen receptor (AR) was not detected in the testis, but estrogen receptor beta (ERβ) was strongly positive in germ cells during the immature period, suggesting that estrogen may regulate spermatogenesis via a paracrine mechanism [34].

The use of the East Asian finless porpoise as the primary model for the Yangtze finless porpoise is supported by their taxonomic affinity: both are recognised as subspecies of *Neophocaena asiaeorientalis*—the Yangtze finless porpoise as *N. a. asiaeorientalis* and the East Asian finless porpoise as *N. a. sunameri*. This close phylogenetic relationship provides a reasonable basis for expecting broad similarities in reproductive physiology, including testicular development, hormonal regulation, and the timing of sexual maturation. However, the validity of this extrapolation is not without limitations. First, the two subspecies occupy markedly different habitats freshwater (Yangtze River and its connected lakes) versus marine (Yellow/Bohai Sea) and environmental factors such as water temperature, salinity, and photoperiod may influence reproductive seasonality and endocrine profiles in ways that are not yet fully understood. Second, direct comparative studies between the two subspecies are scarce, and most of the available data on the Yangtze finless porpoise come from a limited number of captive individuals or post-mortem specimens, which may not fully represent the reproductive physiology of wild populations. Third, while the testicular developmental milestones documented in the East Asian finless porpoise offer valuable reference points, their precise correspondence to those of the Yangtze finless porpoise remains to be confirmed through direct investigation. Therefore, while the East Asian finless porpoise currently represents the best available model for informing cryopreservation protocol development for its critically endangered counterpart, findings from this model should be applied with appropriate caution, and whenever possible, validated against data obtained directly from the Yangtze finless porpoise.

These interspecific differences underscore that a semen cryopreservation protocol for the Yangtze finless porpoise must be tailored to the physiological characteristics of the target species through customised research, with particular attention to species-specific seasonal patterns of sperm production and the hormonal milieu that may influence sperm membrane integrity and cryotolerance.

Compared with marine cetaceans, research on the male reproductive system of freshwater cetaceans is extremely limited. For the Yangtze finless porpoise (*N. asiaeorientalis asiaeorientalis*), a freshwater cetacean endemic to China, relevant anatomical and physiological data remain scarce, and existing studies have mainly relied on non-invasive fecal steroid analysis. In recent years, studies based on necropsy of deceased individuals and ultrasound monitoring of captive animals have gradually revealed the basic characteristics of the male reproductive system of the Yangtze finless porpoise (Figure 2B shows the male reproductive organs of a Yangtze finless porpoise).

Morphological and ultrasonographic observations have shown that the testes of the male Yangtze finless porpoise are located in the abdominal cavity, adjacent to the kidneys. They are long and cylindrical, with a smooth, intact capsule and homogeneous parenchymal echogenicity, mainly composed of the capsule, gonadal parenchyma, and mediastinum testis. There is no significant difference in size between the left and right testes, with the left testis accounting for approximately 52.5–52.64% of the total testicular weight [36,37]. Total testicular mass is significantly positively correlated with body length and body weight, and increases notably after sexual maturity [37]. The gonadal development of the Yangtze finless porpoise can be divided into three stages: juvenile, pubertal, and sexually mature. During puberty, gonadal volume and echogenicity show the first rapid increase. After sexual maturity, the mean gonadal volume during the breeding season reaches 812.57 ± 211.65 cm^3^, with an echogenicity of 103.50 ± 24.01, both significantly higher than during the non-breeding season [36]. Gonadal size continues to increase slowly after initial sexual maturity [38].

Histological and endocrinological studies have further revealed seasonal reproductive characteristics. Based on the staging of testicular development, the process can be divided into early embryonic, late embryonic, premature, and mature stages. When body length reached ≥147 cm, testicular weight increased approximately 14-fold compared with the premature stage, seminiferous tubule diameter increased markedly, and free spermatozoa appeared, indicating sexual maturity. The mature testis exhibited distinct seasonal alternation between active and inactive phases; during the inactive phase, seminiferous tubules atrophied and the tunica albuginea became fibrotic. There was no significant difference in size between the left and right testes (the left testis accounting for 52.5%). The field sex ratio (female:male) was approximately 3:1, which, together with the large testis size, suggests that the Yangtze finless porpoise is a polygynous species, with the large testis of males facilitating the provision of sufficient spermatozoa for mating with multiple females [39]. Serum hormone levels in spring (March–April) showed that testosterone in sexually mature males ranged from 2.64 to 9.40 ng/mL and declined sharply when health deteriorated; estradiol was undetectable in immature females; progesterone in pregnant females reached as high as 30.59–41.19 ng/mL (consistent with late pregnancy levels in the bottlenose dolphin), indicating that progesterone can serve as a diagnostic indicator of pregnancy. Based on the timing of parturition (late April to late May), the mating season was inferred to be from June to August, and the spring testosterone peak did not coincide with the mating season [40]. Furthermore, the diameter of the seminiferous tubules in the Yangtze finless porpoise exhibits pronounced seasonal variation. During the breeding season (March–July), the mean seminiferous tubule diameter in adults (115.75 ± 66.77 μm) is significantly larger than that during the non-breeding season (November and January: 79.2 ± 28.61 μm) [38]. In sexually mature individuals, the seminiferous tubule diameter further increases to 179.43–326.3 μm, with lumina and spermatozoa appearing [37]. This structural pattern is consistent with the findings of fecal hormone monitoring, which indicated that the active reproductive period for males extends from March to September each year, with fecal testosterone concentrations significantly higher during the breeding season (mean approximately 20,060.8 ng/g) than during the non-breeding season (approximately 1232.0 ng/g) [41]. Together, these findings confirm the strongly seasonal nature of reproductive activity in the Yangtze finless porpoise. Table 1 summarizes the key parameters and methodologies of the studies reviewed above.

### 3.2. Macroscopic and Microscopic Characteristics of Semen

Spermatozoa consist of a head (containing the haploid nucleus and acrosome) and a flagellum (comprising midpiece, principal piece, and terminal piece) connected by the neck [42,43,44,45,46]. As these basic features are well established, this section focuses on species-specific variations and their functional implications.

Cetacean sperm heads are flattened and ovoid with a distinct acrosomal ridge, showing significant interspecific variation. Midpiece mitochondria are round and randomly arranged (unlike the helical arrangement in other mammals), and the equatorial segment of the acrosome is absent in cetaceans [47,48].

From a cryobiological perspective, these reproductive and semen characteristics are not merely descriptive. Seasonal endocrine status, epididymal maturation, sperm ultrastructure, membrane composition, mitochondrial organization, and osmotic regulation may jointly determine the intrinsic cryoresistance of cetacean spermatozoa. In particular, sperm collected at different reproductive stages or seasons may differ in membrane lipid composition, membrane fluidity, aquaporin-mediated water transport, mitochondrial activity, and susceptibility to reactive oxygen species. These factors directly influence water efflux, cryoprotectant permeability, osmotic injury, ice crystal formation, and post-thaw motility. Therefore, linking reproductive physiology with functional cryobiological traits is essential for developing species-specific semen cryopreservation protocols.

#### 3.2.1. Differences in Semen Volume and Concentration

Semen macroparameters vary considerably among toothed whales (Table 2). Sperm concentration in the Yangtze finless porpoise (4.17 × 10^9^/mL [49]) appears notably higher than in the Pacific white-sided dolphin (6.20 × 10^8^/mL) and killer whale (1.20 × 10^8^/mL) [50]. Ejaculate volume ranges from 18 mL (bottlenose dolphin [50]) and 12 mL (Pacific white-sided dolphin [50]) to only 1 mL (killer whale), likely reflecting species body size, collection conditions, and individual variation. Collection method strongly affects quality: behaviourally trained live animals yield the highest quality samples (total motility ~90%), whereas post-mortem samples show considerably reduced quality (e.g., beluga: 40% motility after illness and medication) [50]. A bottlenose dolphin study established a transferable cryopreservation protocol (20% egg yolk, 11% lactose, 4% glycerol; pellet freezing on dry ice; −176 °C storage), achieving 80% post-thaw motility [50].

Cross-species comparisons must be interpreted cautiously, as semen characteristics are influenced by numerous uncontrolled confounders: age (ontogenetic changes), reproductive season (particularly in seasonal breeders [41]), ejaculation frequency, collection technique (electro-ejaculation vs. voluntary vs. post-mortem), sexual stimulation, and captive management (diet, temperature, social structure) [38]. These factors may confound apparent interspecific differences, and future studies should adopt standardised protocols and longitudinal within-species sampling.

#### 3.2.2. Differences in Sperm Morphology, Motility, Viability, and Survival

**Figure 3:**
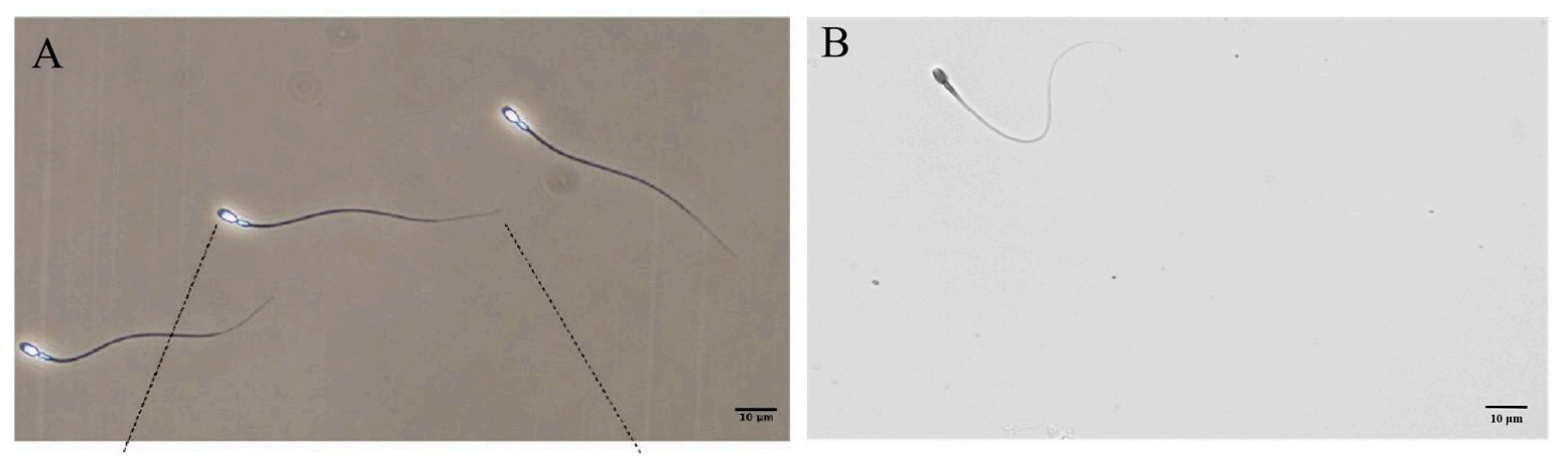
Spermatozoa of bottlenose dolphin (**A**) [48] and Yangtze finless porpoise (**B**) showing the head and flagellum. (**A**) Phase-contrast microscopy, scale bar = 10 µm; (**B**) Light microscopy, scale bar = 10 µm. These morphological characteristics provide the basis for routine sperm quality assessment before and after cryopreservation.

Cetacean spermatozoa exhibit rich interspecific ultrastructural variation (Table 2, Figure 3): (1) Head morphology: The killer whale head is nearly square/paddle-shaped (width 3.3–4.0 μm, ~2× that of the Yangtze finless porpoise at 1.90 μm); Pacific white-sided dolphin and bottlenose dolphin have elliptical heads [50,51]; the Yangtze finless porpoise head is pear-shaped in lateral view [49]. (2) Post-acrosomal structures: Bottlenose dolphin and Pacific white-sided dolphin possess 14–16 longitudinal ridges, proposed to participate in sperm-oolemma fusion; killer whale lacks ridges but exhibits periodic electron-dense bands (~30 nm spacing); no special structures are observed in the Yangtze finless porpoise [49,50,51]. (3) Midpiece mitochondria: Bottlenose dolphin shows two electron-density types (A and B); beluga has a helical sheath with 70–80 turns; Pacific white-sided dolphin and killer whale display random and layered arrangements, respectively; the Yangtze finless porpoise has 4–5 layers with no special arrangement [49,50,51].

#### 3.2.3. Cryobiological Relevance of Cetacean Sperm Ultrastructure

The ultrastructural features described above have direct implications for sperm cryopreservation—a critical tool for endangered cetacean conservation.

Mitochondrial organisation affects cryoinjury susceptibility. The beluga’s extensive helical sheath may enhance mechanical stability but increases membrane surface area vulnerable to ice damage. Simpler arrangements (Pacific white-sided dolphin, killer whale) may reduce cryodamage by facilitating uniform water efflux. The Yangtze finless porpoise (4–5 layers, no special arrangement) represents an intermediate phenotype. Species with complex mitochondrial architecture may require slower freezing rates and higher cryoprotectant concentrations.

Membrane lipid composition is a principal determinant of cryoresistance. The cholesterol-to-phospholipid ratio governs membrane fluidity and stability: higher ratios generally enhance post-thaw survival by maintaining the liquid crystalline phase during cooling. Polyunsaturated fatty acids (PUFAs), particularly DHA and arachidonic acid, are essential for membrane fluidity and fertilising competence but are highly susceptible to oxidative damage. Cryopreservation amplifies ROS production, and species with higher PUFA content may require stronger antioxidant supplementation (e.g., vitamin E, glutathione, catalase). Membrane fluidity also determines osmotic tolerance the ability to withstand volume changes during freeze–thaw cycles and dictates optimal cooling rates and cryoprotectant concentrations, which cannot be simply transferred between species.

Acrosomal and membrane architecture influence cryoprotectant permeability and ice nucleation. The absence of an equatorial segment in cetaceans (unlike most mammals) may fundamentally alter osmotic responses and cryoprotectant uptake. Longitudinal post-acrosomal ridges (bottlenose dolphin, Pacific white-sided dolphin) increase membrane surface area, potentially affecting water permeability and cryoprotectant kinetics.

For the Yangtze finless porpoise, establishing a reliable cryopreservation protocol is an urgent conservation priority. No systematic data exist on its sperm membrane lipid composition, cholesterol/phospholipid ratio, PUFA profile, or osmotic tolerance—information essential for rational protocol design. These functional biochemical investigations should be prioritised alongside descriptive morphological characterisation.

Based on these findings, tentative observations may be suggested (Table 2): (1) Sperm size vs. body weight: Limited data (four species from three families) suggest a possible positive relationship—killer whale > beluga > Pacific white-sided dolphin > Yangtze finless porpoise [49,50]—but this trend requires confirmation with a broader dataset, as it may be confounded by phylogeny and methodology. If confirmed, it would contrast with the negative correlation hypothesis proposed by Cummins and Woodall (1985) at the mammalian level [52]. (2) Acrosome proportion reflects phylogeny: within Delphinidae, acrosome covers ~1/2–3/5 of the head (bottlenose dolphin, Pacific white-sided dolphin) vs. ~3/4 (killer whale); the Yangtze finless porpoise (Phocoenidae) has ~1/2 coverage, similar to delphinids; the beluga (Monodontidae) resembles the killer whale [49,50,51].

Cetacean sperm motility is standardised using three microscopy indices: total motility (TM), percentage of progressive motile sperm (p.p.m.), and kinetic rating (KR, 0–5 scale) [49,50]. Functionally, bottlenose dolphin spermatozoa were shown to require 2 h pre-incubation in Ca^2+^-free medium to acquire oocyte fusion ability (maximum penetration rate 57.7% in zona-free hamster oocytes), with characteristic hyperactivated motility observed the first functional evidence of capacitation in cetaceans [51]. However, heterologous IVF results must be interpreted cautiously: species-specific gamete fusion mechanisms (IZUMO1/JUNO/CD9), bypassing of the zona pellucida (a critical species-selective step), binary readout (fusion vs. no fusion) that does not assess progressive motility, acrosomal exocytosis, or embryonic development support, and weak correlation with in vivo fertility all limit predictive value. For endangered species like the Yangtze finless porpoise, heterologous assays remain useful for initial characterisation but should be interpreted alongside other functional markers (motility, membrane integrity, acrosomal status) rather than as definitive fertility predictors.

## 4. Semen Collection Techniques and Challenges in Cetaceans

Semen collection is foundational for cetacean reproductive biology and germplasm conservation, yet poses multiple technical, operational, and ethical challenges. This section critically evaluates three principal methods regarding reproducibility, sample quality, animal welfare, and applicability to endangered species. Collection method directly influences sperm motility, membrane integrity, contamination, and cryosurvival, and must be considered integral to the biobanking pipeline.

### 4.1. Electroejaculation

EEJ has been reported in only one successful case in cetaceans [50], and its application remains extremely limited because of concerns regarding sample quality, animal welfare, and ethical acceptability. Although EEJ may provide an alternative when voluntary semen collection is impossible, its application in cetaceans remains constrained by methodological, biological, and ethical limitations. To date, no controlled within-species study has quantitatively compared sperm quality obtained by electroejaculation and voluntary collection in cetaceans. Consequently, current recommendations are primarily based on limited cetacean reports and comparative evidence from other mammalian species. From an ethical perspective, EEJ generally requires anaesthesia or deep sedation and has the potential to induce substantial stress and discomfort. Accordingly, approval by institutional animal ethics committees is generally restricted to exceptional circumstances, such as when the only fertile male cannot be trained and no feasible alternative exists. Justifiable use further requires that the individual is untrainable, the genetic resource is irreplaceable, the procedure is performed by experienced personnel, and appropriate ethical approval has been obtained. A comprehensive comparison between EEJ and voluntary semen collection is provided in Table 2 (Section 4.4). Given these limitations, EEJ should not be considered a routine method, and its application should be restricted to exceptional cases where no feasible alternative exists.

### 4.2. Voluntary Collection via Positive Reinforcement Training (PRT)

PRT combined with operant conditioning is the gold standard for trainable odontocetes [33]. Males are trained in dorsal recumbency at the pool edge, with tactile stimulation inducing penile extrusion and ejaculation into a sterile bag; wiping, rinsing, or an isolation ring minimises seawater contamination. PRT offers clear advantages, including excellent welfare (voluntary, low stress), high repeatability (long-term regular collections), and good quality when contamination is controlled. However, PRT efficiency is species- and individual-dependent; training ranges from 6–10 weeks to 2 years in bottlenose dolphins, whereas nearly 3 years were needed for the timid Yangtze finless porpoise. The method relies on experienced trainers and is inapplicable to wild populations or short-term rescues. Regarding welfare, voluntary participation minimises stress, and cortisol is a reliable indicator; although no study has directly measured cortisol during PRT collection, PRT has been shown to reduce handling stress generally. Long-term effects on sperm traits lack data, and future work should combine behavioural scoring with non-invasive hormone monitoring. In 2025, the Wuhan Baiji Dolphinarium achieved successful PRT collection from a 7-year-old male within seconds, demonstrating proof of concept, though long training periods and individual variability remain barriers.

### 4.3. Post-Mortem Epididymal Sperm Recovery

When a valuable individual dies, testicular or epididymal tissue collection offers a last-chance strategy for genetic resources. Sperm quality declines rapidly post-mortem; necropsy beyond 38–56 h causes irreversible damage, and even short intervals (<6 h) with agonal heat stress impair sperm membranes and DNA integrity [53]. No cetacean-specific maximum interval is defined; recovery should be as rapid as possible. Refrigeration at 4–5 °C is recommended for temporary storage and transport. Regarding DNA integrity, Spanish ibex studies showed no significant differences at 2–24 h post-mortem, enabling ICSI/IVF even with delayed processing, but mitochondrial membrane potential declines after 12 h, reducing motility and increasing osmotic sensitivity [54]. Rapid freezing causes less membrane and DNA damage than slow freezing but may worsen apoptosis [53]. Cryopreservation protocols differ: epididymal sperm lack seminal plasma exposure, whereas ejaculation induces plasma membrane maturational changes. Epididymal sperm require shorter cryoprotectant equilibration, have different membrane and osmotic properties, and need exogenous cryoprotectants to compensate for absent seminal plasma; protocols cannot simply adopt those for ejaculated sperm. For the Yangtze finless porpoise, a rapid-response workflow is recommended: immediately transfer intact testes (with epididymides) at 4–5 °C, avoiding direct ice contact, delivering within 24 h; aseptically isolate the cauda epididymidis (highest progressive motility [55]), removing fat and blood; complete extraction and freezing within 6 h, as beyond 12 h mitochondrial function is impaired [54]; use rapid freezing [53] and TEST-yolk or soy lecithin buffers to compensate for absent seminal plasma, with equilibration shorter than for ejaculated protocols.

### 4.4. Method Comparison and Conservation Application Recommendations

The three methods differ substantially in sperm quality, welfare, feasibility, and cost (Table 3).

The impact on cryosurvival is multi-faceted. Seminal plasma composition differs: native in PRT, altered in EEJ, and absent in epididymal sperm (requiring exogenous protectants). Stress effects: EEJ elevates cortisol, damaging membranes, while PRT minimises this but requires adequate recovery. Contamination: seawater and urine (PRT) as well as blood and debris (post-mortem) reduce survival unless washed. Repeatability: PRT enables longitudinal assessment, whereas post-mortem and EEJ are one-off. Therefore, endangered programmes should prioritise PRT where feasible, establish post-mortem as backup, avoid EEJ, and validate method-specific extenders and cooling curves.

For the Yangtze finless porpoise specifically, captive populations are small. PRT has achieved initial success but faces challenges due to timid temperament. Recommendations include accelerating PRT with dedicated behaviourists, developing standardised post-mortem collection kits for stranding or mortality events, conducting comparative studies to tailor species-specific freezing protocols, and establishing a central database to guide best practices. The choice of semen collection method is not merely technical but a strategic decision affecting welfare, quality, and conservation success. A balanced, welfare-oriented, and method-specific approach will maximise the long-term conservation value of semen collection while safeguarding animal welfare and genetic diversity.

Because cetaceans, particularly endangered species such as the Yangtze finless porpoise, are protected wildlife, all semen collection procedures should be conducted under appropriate governmental permits, institutional animal ethics approval, and the supervision of experienced veterinarians. Animal welfare should remain the primary consideration throughout semen collection and assisted reproductive procedures. Live-animal semen collection should be undertaken only when the expected conservation benefits clearly outweigh the potential welfare risks and when no less invasive alternative is available. Consequently, the routine use of invasive techniques in endangered cetaceans cannot be justified and should be reserved for exceptional circumstances in which clear conservation benefits are anticipated. Accordingly, semen collection should adhere to the principles of Replacement, Reduction, and Refinement (the 3Rs), with preference given to non-invasive or minimally invasive approaches whenever feasible.

## 5. Advances in Core Technologies for Cetacean Semen Cryopreservation and Thawing

**Figure 4:**
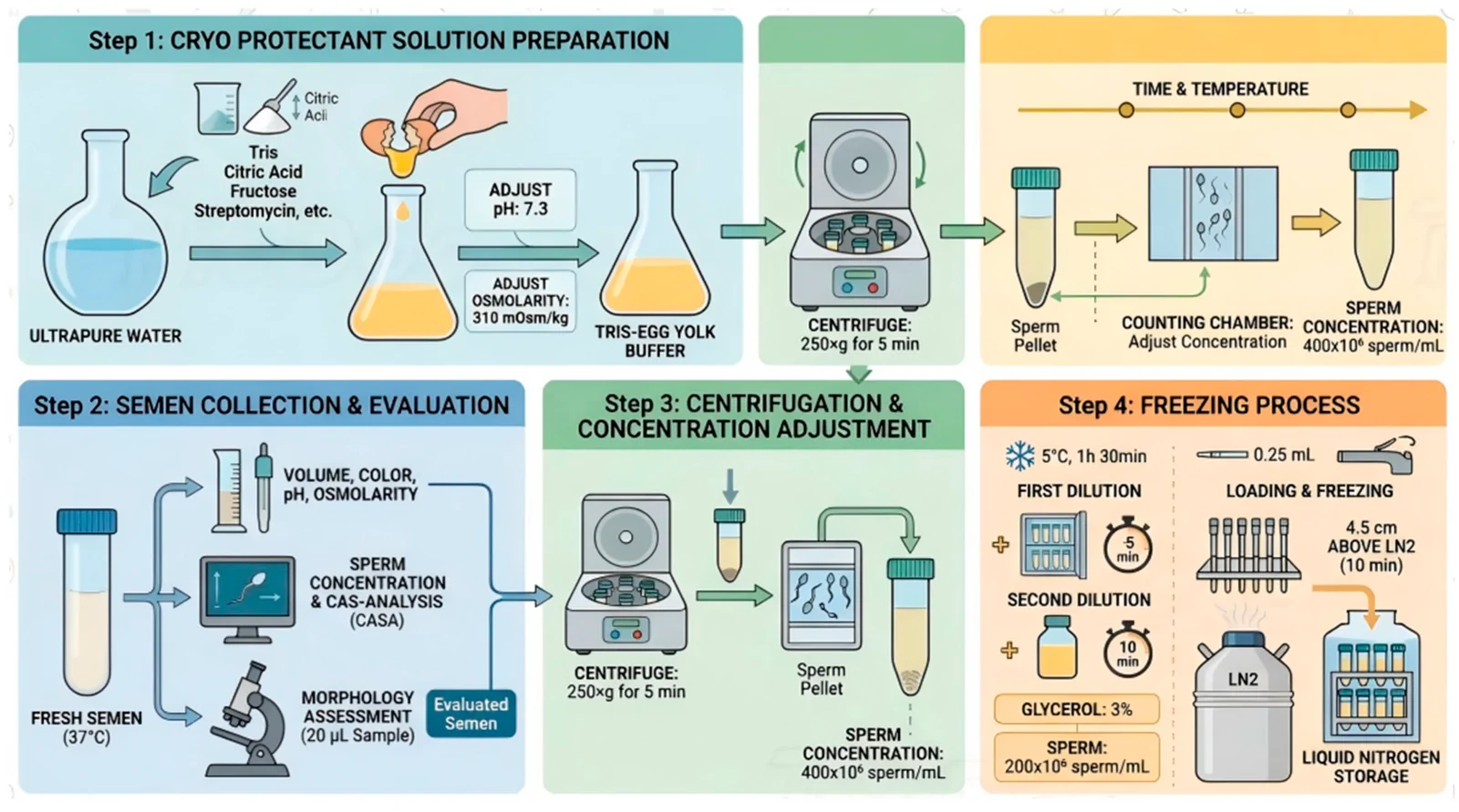
Schematic diagram of the semen cryopreservation process.

Significant progress has been made in the cryopreservation of semen from the bottlenose dolphin, Pacific white sided dolphin, and killer whale, establishing a complete technical system encompassing semen collection, processing, freezing, thawing, and post thaw assessment (Figure 4). These advances have laid a solid foundation for the conservation of cetacean germplasm resources and offer potential for assisted reproduction in endangered species.

### 5.1. Semen Initial Processing and Assessment

Generally, semen is subjected to centrifugation and dilution immediately after collection. The considerable variation in centrifugation protocols among studies complicates direct comparisons of cryopreservation outcomes and may contribute to differences in post-thaw sperm quality; for the bottlenose dolphin, reported conditions range from 250× *g* to 5000 rpm, and post-dilution sperm concentration is often adjusted to 2–4 × 10^8^/mL [56,57,58]. Nevertheless, the potential impact of these centrifugation parameters on sperm quality has not been critically evaluated in the studies reviewed. Centrifugation force and duration are known to influence sperm membrane integrity, induce oxidative stress, cause acrosomal damage, and promote DNA fragmentation [59,60,61], all of which could confound post-thaw assessments. Consequently, differences in post-thaw outcomes reported across studies may partly reflect variations in initial semen processing rather than the efficacy of cryopreservation protocols themselves. Future studies should consider standardizing centrifugation conditions or, at minimum, reporting detailed processing parameters to enable more meaningful cross-study comparisons. For the Pacific white sided dolphin and killer whale, centrifugation conditions are less frequently reported, but immediate post collection assessment is consistently emphasized [62,63].

For motility assessment, computer assisted sperm analysis (CASA) is predominantly used in the bottlenose dolphin [56,57,58], whereas subjective evaluation was primarily relied upon in early studies of the Pacific white sided dolphin and killer whale [62,63]; later studies on killer whales gradually adopted CASA [63]. To improve accessibility for readers outside reproductive biology, a summary table defining the principal CASA variables (VAP, VSL, VCL, LIN, STR, ALH, and BCF) is provided in Table 4. Fresh semen quality is generally high: in the bottlenose dolphin, total motility exceeds 84%, with progressive motility reaching up to 84.5% [57,64]; in the Pacific white sided dolphin, the sperm motility index (SMI) ranges from 434 to 467, with progressive motility >87% [62]; in the killer whale, fresh semen exhibits total motility as high as 92.2%, progressive motility of 85.4%, and CASA parameters of VAP 259 μm/s and VCL 316 μm/s [63]. In addition, the sperm chromatin dispersion test (SCDt) has been applied in bottlenose dolphin studies to assess DNA fragmentation, with the sperm fragmentation index (SFI) in fresh semen being extremely low (0.7–2.0%) and remaining stable within 48 h of incubation at 37 °C [58]. Notably, this finding is based on a single study, and the current evidence base for DNA integrity assessment in cetaceans remains limited; further studies across more species and cryopreservation protocols are needed. Beyond cryopreservation protocols, ambient physicochemical conditions such as salinity and pH have also been shown to significantly influence bottlenose dolphin sperm viability [61], further emphasizing the importance of optimizing extender conditions for this species.”

Additionally, CASA settings frame rate, particle size, and velocity thresholds vary across laboratories and can significantly affect reported values. In the studies reviewed, CASA protocols were not standardized; only one study on killer whales reported specific settings (60 Hz frame rate, minimum contrast 60, minimum cell size 15 pixels, VAP > 20 μm/s for progressive motility, STR > 70%) [63], whereas bottlenose dolphin studies either used subjective assessment or did not specify settings [56,57,58]. Therefore, direct comparisons of motility parameters across studies should be made with caution.

### 5.2. Selection and Optimization of Cryoprotectants

Cryoprotectants (CPAs) exert two opposing effects during cryopreservation: protection against intracellular ice formation and inherent chemical toxicity. Glycerol remains the most widely used penetrating CPA in cetacean semen cryopreservation, but its protective efficacy is concentration-dependent and must be carefully balanced against cytotoxicity. Glycerol toxicity may occur through several mechanisms, including disruption of enzymatic activity, destabilization of the cytoskeleton, and alterations in intracellular pH and osmotic gradients. In addition, both the addition and, particularly, the removal of glycerol during thawing impose substantial osmotic stress, as rapid water and glycerol fluxes may cause excessive cell shrinkage, swelling, or membrane rupture [61,65,66,67,68].

Despite these risks, the optimal glycerol concentration varies among species. Effective concentrations of 3–7% have been reported for the bottlenose dolphin [56,57,58,64], 5–6% for the Pacific white-sided dolphin [62], and 3–6% for the killer whale, with 6% yielding the best post-thaw results in later optimization studies [33,63]. The mechanistic basis for these interspecific differences in glycerol tolerance is likely multifactorial. First, variation in plasma membrane lipid composition, particularly the degree of fatty acid unsaturation and cholesterol content, affects membrane fluidity and permeability, thereby influencing glycerol transport across the bilayer and intracellular accumulation [69,70,71,72]. Second, differential expression and activity of aquaporins, especially AQP3 and AQP7, regulate transmembrane water and glycerol movement, influencing both CPA equilibration kinetics and the duration of cytotoxic exposure [19,73]. Third, species-specific osmotic tolerance limits, governed by volume-regulatory ion channels and organic osmolyte transporters, determine the ability of spermatozoa to withstand the substantial volume changes induced by CPA addition and removal [74,75,76,77]. Together, these interacting factors define a species-specific “safe window” for glycerol concentration.

Non-penetrating cryoprotectants and membrane protectants are also important components of cetacean sperm extenders. In the protocols reviewed, 20% egg yolk is commonly incorporated as a basal membrane protectant, while bottlenose dolphin extenders have also been supplemented with sugars such as glucose, lactose, and trehalose, as well as glutathione and antibiotics [56,57,78]. Egg yolk-free formulations, including LP1 and HSPM, have demonstrated adequate membrane protection and maintenance of post-thaw motility; however, LP1 was associated with significantly increased DNA fragmentation after prolonged incubation [58].

This finding warrants further mechanistic consideration. LP1 is a soybean lecithin-based extender, whereas HSPM is formulated for human clinical use. The lipid profile of plant-derived lecithin differs substantially from that of egg yolk or natural seminal plasma, particularly in cholesterol and specific phospholipid fractions that contribute to sperm membrane stabilization during freeze–thaw stress. Egg yolk-derived lipoproteins and phospholipids, including phosphatidylcholine and sphingomyelin, are important for maintaining sperm membrane fluidity and integrity [73,79]. Their absence in egg yolk-free formulations may reduce the membrane’s buffering capacity against freeze–thaw stress and increase susceptibility to oxidative phospholipid damage [80,81,82]. Because membrane integrity loss has been significantly associated with DNA fragmentation in multiple studies [83,84], reduced membrane protection may provide a plausible pathway linking extender composition to delayed chromatin damage.

In a study investigating post-thaw DNA fragmentation dynamics, spermatozoa cryopreserved in LP1 showed significantly higher DNA fragmentation after 24 h of incubation, although no differences were detected among TTF, LP1, and HSPM immediately after thawing (T0) [58]. The authors proposed that LP1 may provide less effective membrane protection than egg yolk-based extenders, making the plasma membrane more vulnerable to damage during subsequent incubation and thereby promoting ROS-mediated DNA fragmentation. Although both LP1 and HSPM are animal protein-free formulations and therefore offer potential advantages in reducing the risk of disease transmission, LP1 was less effective than TTF and HSPM in maintaining DNA integrity during prolonged incubation. These findings indicate that extender efficacy should not be evaluated solely on the basis of immediate post-thaw sperm quality, but should also include DNA integrity assessment after incubation, which may better reflect delayed cryo-induced damage.

These findings have important implications for conservation breeding. Genome resource banking and artificial insemination are vital for the genetic management of ex situ cetacean populations and for the conservation of endangered small cetaceans. Because sperm DNA integrity is a critical determinant of fertilization and embryo development, an extender that preserves motility but permits delayed DNA fragmentation may lead to overly optimistic assessments of post-thaw sperm quality while compromising actual fertility. Therefore, extender selection should not rely solely on motility; DNA integrity after incubation, which may better simulate post-insemination intervals, should be incorporated as a key evaluation criterion.

Whether fertilization outcomes, rather than motility alone, have been compared among different cryoprotectants or extenders in cetaceans remains an important question. Homologous in vitro fertilization (IVF) is technically difficult because of limited oocyte availability. However, heterologous IVF using bovine oocytes has been developed as a surrogate assay for evaluating dolphin sperm fertilizing capacity, demonstrating that cryopreserved dolphin sperm can penetrate bovine oocytes and form hybrid embryos. Nevertheless, systematic comparisons of fertilization outcomes among different extenders, such as LP1, HSPM, and egg yolk-based formulations, have not yet been reported. This remains a critical knowledge gap and should be prioritized in future research, as fertilization-based endpoints would provide more direct evidence of functional fertility preservation. Finally, in the killer whale, a systematic comparison of BF5F, Biladyl^®^, and EYC showed that BF5F, which contains TES, Tris, glucose, fructose, and egg yolk, was the most effective [82]. This finding further reinforces the importance of empirical, species-specific screening of CPA concentration and extender composition.

### 5.3. Extender Formulation and Osmolality Selection

Extender formulations used for cetacean semen cryopreservation show several common features across species, typically containing Tris, citric acid, fructose or glucose, and 20% egg yolk as major components (Table 5). In bottlenose dolphins, the Reagent I/II formulation had an osmolality of 345 ± 5 mOsm/kg [56], whereas other studies maintained extender osmolality within the range of 310–330 mOsm/kg [57,64,80]. In the killer whale, seminal plasma osmolality was reported to be 359 mOsm/kg with a pH of 7.4 [63], providing a potential reference for extender design. However, direct extrapolation of this value to other cetaceans should be approached with caution, because seminal plasma composition may vary substantially among species as a result of differences in reproductive physiology and accessory gland function. For example, BF5F used for Pacific white-sided dolphin semen had an osmolality of 330 ± 5 mOsm/kg and a pH of 7.0 [80], further suggesting the need for species-specific adjustment of extender formulation.

Osmolality plays a central role in cryopreservation because it regulates water movement across the sperm plasma membrane, thereby influencing cell volume, cryoprotectant equilibration, and the risk of intracellular ice formation [21]. Under hyperosmotic conditions, water efflux induces cellular dehydration and shrinkage, reducing the amount of freezable intracellular water and lowering the risk of lethal ice crystallisation during freezing [85]. In contrast, hypo-osmotic conditions promote water influx, sperm swelling, increased membrane tension, and potential membrane rupture or permeability dysfunction [86,87]. The osmotic gradient between the intra- and extracellular compartments also affects cryoprotectant permeation. An excessively steep gradient may cause rapid cell shrinkage and osmotic shock, whereas an insufficient gradient may prolong equilibration and increase exposure to CPA toxicity [18,88,89]. Therefore, extender osmolality must be optimized to balance dehydration, cryoprotectant permeability, osmotic tolerance, and membrane stability.

To date, no cetacean study has directly compared post-thaw sperm quality using extenders with osmolalities matched or mismatched to species-specific seminal plasma. Until such empirical data become available, extender osmolality design should be guided by seminal plasma measurements, while also incorporating systematic comparisons of post-thaw sperm quality across a defined osmolality range. Such studies would help determine whether seminal plasma osmolality provides an appropriate target value or whether cryopreservation requires a deliberately modified osmotic environment to improve sperm survival.

### 5.4. Development and Evaluation of Freezing Protocols

Freezing protocols vary among cetacean species and are strongly influenced by the equipment, packaging format, and cooling strategy used. In bottlenose dolphins, reported methods include stepwise cooling, liquid nitrogen vapour freezing, and programmable freezing, with cooling rates ranging from −0.27 °C/min to −100 °C/min [56,57,58,64]. In Pacific white-sided dolphins, sperm has been cryopreserved using straw-based vapour freezing at −0.27 °C/min and directional freezing at −0.2 °C/min with induced ice nucleation [80]. In killer whales, straw-based cooling rates of −10.7 to −14.6 °C/min under SLOW and MEDIUM conditions produced better results than the faster rate of −15.2 °C/min [60]. Directional freezing, in which samples are moved through a −50 °C temperature gradient at 1 mm/s, has also shown promising results in limited studies, enabling controlled linear ice propagation and yielding superior post-thaw sperm quality compared with straws within the same experimental study [63].

Cooling rate is a critical determinant of sperm survival because it governs the balance among osmotic stress, intracellular ice formation, and membrane integrity [90]. Excessively slow cooling prolongs water efflux, resulting in severe cellular dehydration and increased intracellular solute concentration, both of which can destabilize sperm membranes. Conversely, overly rapid cooling may leave residual freezable water inside the cell, increasing the risk of lethal intracellular ice crystallization. The optimal cooling rate therefore represents a balance between dehydration injury and ice crystal injury, and is largely determined by membrane permeability to water and cryoprotectants [91,92,93].

Packaging format further modulates freezing outcomes by altering heat transfer efficiency, thermal uniformity, and ice nucleation patterns. Common formats include 3 mL cryovials, 0.25 mL and 0.5 mL straws, and 2 mL or 9 mL hollow tubes for directional freezing [56,57,58,62,63,69,70,71,72,73,74,75,76,77,78,79,80,81,82,83,84]. Cryovials have relatively large volumes and thick walls, which may result in slower and less uniform cooling, with ice nucleation often occurring at the periphery [56,58]. Straws, by contrast, have higher surface-area-to-volume ratios and therefore allow faster and more homogeneous heat transfer; 0.25 mL straws generally cool more rapidly than 0.5 mL straws [57,63]. Directional freezing tubes impose a controlled linear temperature gradient, allowing ice nucleation and propagation to be regulated more precisely and enabling more uniform freezing of larger-volume samples [63,80].

Nevertheless, the studies reviewed differ not only in cooling rate, but also in extender composition, cryoprotectant type and concentration, packaging format, thawing procedure, and baseline semen quality. Direct quantitative comparisons should therefore be interpreted with caution, because observed differences in post-thaw outcomes may reflect interactions among multiple variables rather than cooling rate alone. Future studies should compare freezing methods under standardized conditions within the same experimental design, using consistent extenders, cryoprotectant concentrations, packaging formats, thawing procedures, and sperm assessment endpoints. Such studies would help clarify species-specific optimal protocols and strengthen the evidence base for cetacean sperm cryopreservation.

### 5.5. Standardization of Thawing Protocols

Cetacean semen thawing generally follows the principle of rapid warming, although specific thawing conditions vary among species, packaging formats, and freezing methods. In bottlenose dolphins, thawing was commonly performed at 35–37 °C, with durations ranging from 30 s to 2 min [56,57,58,64]. In Pacific white-sided dolphins, straws were thawed at 35 °C for 1 min, whereas directional freezing tubes were first warmed in air for 90 s and then transferred to a 35 °C water bath [80]. In killer whales, straws were thawed at 35 °C for 30 s, corresponding to a warming rate of 8.3 °C/s, while directional freezing tubes were thawed in air for 45 s followed by 45 s in a specialised 35 °C water bath, with an estimated warming rate of 171 °C/min [33,63]. In some studies, post-thaw dilution or density gradient centrifugation was subsequently applied to remove cryoprotectants, dead spermatozoa, and cellular debris [57]. However, although these procedures may improve laboratory sperm quality parameters, no cetacean study has yet demonstrated that post-thaw sperm selection leads to improved fertility outcomes.

The emphasis on rapid warming is biologically justified, as thawing can be as critical as freezing for sperm survival [94]. Slow or uneven warming may promote recrystallisation, a process in which small ice crystals merge into larger crystals that can physically damage intracellular structures [95]. In addition, inappropriate thawing can induce osmotic and thermal stress, resulting in membrane disruption, impaired permeability regulation, and loss of sperm function [21]. These risks are strongly influenced by packaging format and sample volume. Compared with straws, directional freezing tubes have larger volumes and lower surface-area-to-volume ratios, requiring longer and more carefully controlled warming procedures. However, the optimal thawing protocol for directional freezing tubes remains less well defined than that for straws [63].

Future studies should systematically compare thawing temperature, warming rate, thawing duration, and post-thaw handling procedures under standardized experimental conditions. Particular attention should be given to the interaction between packaging format, sample volume, cryoprotectant concentration, and CPA removal strategy, as these factors jointly determine osmotic recovery and membrane stability after thawing. Such work would help distinguish the independent effects of thawing rate from those of associated handling procedures and support the development of more reliable, format-specific thawing protocols for cetacean semen cryopreservation.

### 5.6. Post Thaw Semen Quality Assessment

As summarized in Table 2, post thaw semen quality is the core indicator for evaluating cryopreservation protocols. In the bottlenose dolphin, post thaw survival rates ranged from 32.4% to 84.5%, with significant differences among protocols [56,57,58,64]. In the Pacific white sided dolphin, directional freezing achieved a post thaw total motility of 82.5% and progressive motility of 79.7%, significantly outperforming the straw method [80]. In the killer whale, directional freezing (6% glycerol) yielded a total motility of 75.5% and progressive motility of 56.7% at 3 h post thaw, with VAP of 164.7 μm/s and a combined viability and acrosome integrity of 57.9%, whereas the conventional straw method produced only 50.4% total motility [63].

It is important to recognize, however, that most studies rely heavily on laboratory parameters total motility, progressive motility, and viability which, while useful for assessing sperm survival, may be poor predictors of actual fertilizing capacity. Sperm survival (the ability to remain motile or membrane intact after thawing) is not equivalent to sperm function (capacitation, acrosome reaction, and oocyte penetration), nor to fertilizing capacity (the ability to produce a viable embryo and pregnancy). Spermatozoa that appear viable in standard assays may still be functionally compromised due to subtle membrane damage, mitochondrial dysfunction, or DNA fragmentation. The field would therefore benefit from incorporating more functional assays such as heterologous IVF [57] or AI trials as complementary endpoints.

Regarding DNA fragmentation, the egg yolk free formulation LP1 significantly increased SFI after 24 h of incubation in bottlenose dolphins [58], while a killer whale study suggested that directional freezing may reduce DNA fragmentation [63]. Given the extremely limited evidence confined to a single bottlenose dolphin and one killer whale study DNA integrity assessment should be identified as a priority research area.

For functional assessment, heterologous IVF using bottlenose dolphin spermatozoa validated zona binding and penetration ability of freeze thawed spermatozoa [57]. However, while heterologous assays using hamster or bovine oocytes provide useful experimental models for assessing sperm function, their predictive value for actual fertility in cetaceans remains uncertain, as species specific differences in gamete recognition may not be fully recapitulated in heterologous systems. AI achieved pregnancies across all three species, with pregnancy rates of 67% in the bottlenose dolphin, 50% in the Pacific white sided dolphin, and 38% in the killer whale using frozen thawed spermatozoa [33,64,80]. Although encouraging, these rates should be interpreted cautiously due to the limited number of inseminations performed. Regarding the mini-mum effective dose, the bottlenose dolphin and Pacific white sided dolphin required similar doses (approximately 270 million progressively motile spermatozoa) [64,80]. However, these thresholds derived from single studies and cannot yet be generalized to other odontocetes, as species specific differences in sperm quality, female reproductive tract anatomy, and insemination technique influence the minimum dose required. Establishing minimum effective doses for additional species remains a priority for future research.

Overall, the apparent superiority of one cryopreservation protocol over another should be interpreted cautiously unless the comparison is performed within the same species, using semen from the same or comparable individuals, identical initial processing procedures, standardized CASA settings, the same extender background, controlled cryoprotectant concentrations, equivalent packaging formats, and identical thawing and post-thaw assessment protocols.

## 6. Application of Cryopreserved Semen in Assisted Reproduction and Integrated Conservation Management

### 6.1. Artificial Insemination

Breakthroughs in artificial insemination (AI) using cryopreserved cetacean semen have been achieved in several species. In the bottlenose dolphin, a standardised protocol has been established using a serum estradiol (E_2_) peak ≥ 100 pg/mL to determine insemination timing, combined with intrauterine insemination. Using frozen thawed semen stored long-term in liquid nitrogen, a pregnancy rate of 66.7% has been achieved, with post thaw progressive motility identified as the key factor determining success [60]. In the killer whale, which has a complex cervical structure, hysteroscopically guided intrauterine insemination has successfully produced live offspring [55]. The beluga has also seen the world’s first successful calving following AI with cryopreserved semen [61]. Although these studies demonstrate the feasibility of using cryopreserved semen for artificial insemination, the total number of documented pregnancies remains limited, and routine application across cetacean species has not yet been achieved.

However, while acknowledging these technical advances, it is essential to recognise their limited conservation benefits. First, the number of documented successes is very small, and they are heavily concentrated in a few controlled captive settings. There is currently no evidence that the technique can be reproducibly applied to wild populations or used to reverse species declines.

Second, the high success rates reported in cetacean AI depend on three stringent conditions: (1) precise ovulation timing through frequent serum hormone assays and ultrasonography, which requires extensive animal training and cooperation and is impractical in field or semi-natural settings; (2) the use of specialised equipment such as endoscopes to overcome species-specific cervical anatomical variations (e.g., the tortuous cervix of the killer whale), which increases procedural difficulty, cost, and potential animal stress; and (3) substantial inter-individual and inter-batch variability in post-thaw sperm quality, with no unified quality control system yet established. Moreover, the sperm-dose thresholds currently available are largely based on bottlenose dolphin studies and should not be generalised to other cetacean species without further validation. As a result, the current application of AI in cetaceans remains more of an exceptional research demonstration than a mature conservation tool [27,53,60].

Thus, a considerable gap persists between “technical feasibility” and “routine application in conservation practice” for cetacean AI. Future research should focus on transitioning AI from “successful operations in trained captive individuals” to “standardised, cost-effective, and cross-species protocols”, and systematically evaluate its cost-effectiveness in real-world population management.

### 6.2. Construction and Expansion of Cetacean Genetic Resource Banks

Taking the critically endangered Yangtze finless porpoise as an example, active efforts to establish a germplasm bank have already been initiated as part of ongoing conservation programs. Current work includes the development of positive reinforcement training for voluntary semen collection, cryopreservation of semen and reproductive tissues from captive individuals and opportunistically recovered specimens, and optimization of species-specific cryopreservation protocols. Nevertheless, these initiatives remain at an early developmental stage. To maximize their long-term conservation value, future efforts should progress through three coordinated phases. In the short term, emphasis should be placed on expanding sample acquisition and optimizing semen collection and cryopreservation protocols. In the medium term, standardized procedures should be established, including systematic comparisons of cryoprotectants, extender formulations, and post-thaw quality assessment using CASA, flow cytometry, and DNA integrity assays. In the long term, cryobanked germplasm should be fully integrated into genetic management programs to facilitate gene flow among managed populations, reduce inbreeding, and support assisted reproduction within an integrated conservation management framework.

Current and future cryobanking initiatives should place strong emphasis on long-term genetic management rather than simply increasing the number of stored samples. Germplasm collection should follow a scientifically designed sampling strategy that maximizes founder representation, captures genetic diversity across different management units, and incorporates pedigree and genomic information into donor selection. In this way, cryobanking can function as an effective component of integrated conservation management rather than solely as a repository of frozen biological material.

In terms of genetic representativeness, given the small effective population size and fragmented distribution of the Yangtze finless porpoise, priority should be given to individuals with high genetic contribution potential, low mean kinship, and complementary haplotypes, based on pedigree data and SNP information where available. Founder representation should be balanced across major geographic subpopulations (e.g., Poyang Lake and the main Yangtze mainstream), and samples from unrelated individuals should be prioritised to maximise allelic diversity per sample. Nevertheless, it must be emphasised that even a carefully constructed germplasm bank, without a clear management plan for its use in live population breeding, will have its conservation value remain only on paper. Genetic representativeness of stored samples is a necessary prerequisite, but not a sufficient condition. The ultimate conservation benefit depends on whether stored germplasm can be effectively incorporated into assisted reproduction and long-term genetic management programmes when required.

For critically endangered cetaceans, application of assisted reproductive technologies should always be guided by the principle that conservation benefits must outweigh potential welfare risks. Reproductive interventions should therefore be regarded as complementary conservation measures rather than stand-alone solutions and should be implemented only within ethically approved, scientifically justified, and professionally supervised conservation programmes that are integrated with habitat protection, population management, genetic monitoring, and broader in situ conservation strategies.

In addition to conventional mature sperm cryopreservation, spermatogonial stem cell (SSC) cryopreservation has emerged as a promising complementary pathway. SSCs can be induced to differentiate into functional spermatozoa in vitro or used in testicular transplantation to restore fertility [85,86,87,88,89,90,91,92,93,94,95,96,97,98,99]. Although SSC cryopreservation is well established in mice, its application in cetaceans remains exploratory. Reactive oxygen species accumulation during freeze–thaw is a core issue limiting SSC survival; antioxidant supplementation and optimisation of freeze–thaw rates can mitigate this problem [100]. However, it should be noted that SSC technology is much further from practical application in cetaceans, and its current value lies more as a forward-looking technical reserve than as an immediately usable conservation tool.

Finally, biosecurity represents another critical aspect often overlooked in cetacean cryobanking. Cryobanks may inadvertently preserve pathogens, viruses, or microbial contaminants alongside germplasm. To mitigate risks, comprehensive disease screening of donors, strict quality control during storage, and prevention of cross-contamination should be mandatory components of any cetacean germplasm bank protocol. Failure to address this not only compromises sample quality but may also introduce new health risks in conservation practice, thereby undermining the very purpose of conservation.

## 7. Current Bottlenecks and Future Directions

Although substantial progress has been made in cetacean semen cryopreservation, several critical bottlenecks continue to limit its broader application. Among these, the lack of standardized and reliable criteria for post-thaw sperm functional assessment remains particularly important, as it directly constrains accurate prediction of the true fertilizing potential of thawed spermatozoa. Future research should therefore focus not only on improving cryopreservation protocols, but also on establishing comprehensive evaluation systems and optimizing post-thaw processing strategies to maximize the functional competence of cryopreserved sperm. Although greater methodological standardization would improve comparability among studies, species-specific optimization will likely remain necessary because of the marked biological diversity among cetaceans.

In this section, we synthesize the principal bottlenecks identified throughout this review into four interconnected priorities: (1) addressing species-specific protocol optimization, (2) expanding the taxonomic coverage of cryopreservation studies, (3) developing functional sperm assessment tools, and (4) optimizing post-thaw processing strategies. We further assess the feasibility and potential impact of each priority.

Rather than treating these challenges as isolated issues, we propose that they are hierarchically organized. The primary bottleneck is the severe limitation in taxonomic coverage and sample availability, because without sufficient biological material from diverse species, neither protocol optimization nor assay validation can be robustly pursued. The secondary bottleneck is the lack of species-specific protocols, which is both a consequence of limited sample availability and an independent barrier to standardization. The tertiary bottleneck is the absence of validated functional assessment tools, which in turn limits accurate evaluation of post-thaw processing improvements. This hierarchical perspective informs the structure of the following subsections and the prioritization proposed in Section 7.5.

### 7.1. Acknowledging and Addressing Species-Specific Protocol Variation

The difficulty of establishing standardized protocols across cetaceans is not merely a consequence of methodological inconsistency, but also reflects profound biological diversity among species. Cryopreservation outcomes are influenced by multiple species-specific factors, including sperm membrane lipid composition, seminal plasma biochemical profile, osmoregulatory capacity during freeze–thaw stress, seasonal reproductive physiology, and semen collection method. For example, interspecific differences in the proportion of polyunsaturated fatty acids in the sperm membrane may directly affect susceptibility to cold shock and oxidative damage during cryopreservation. Similarly, seminal plasma composition can vary substantially among species in antioxidant capacity, protein content, and electrolyte balance, all of which may influence the effectiveness of a given cryoprotectant or extender formulation. Consequently, a protocol that achieves high post-thaw survival in one species may be suboptimal, or even detrimental, in another.

Although greater methodological standardization is important for improving comparability among studies, complete protocol standardization across all cetaceans may not be biologically feasible. A more practical strategy is therefore to develop species-specific optimization frameworks while maintaining a set of core endpoints and assessment criteria that enable meaningful cross-species comparisons. This approach is conceptually straightforward and immediately actionable, as it requires a shift in experimental design and reporting practice rather than a major technological breakthrough. However, its successful implementation depends on the continued accumulation of species-specific cryopreservation data, which remains limited for most cetaceans. This limitation cannot be resolved by methodological refinement alone and is directly linked to the broader issue of sample availability discussed in Section 7.2.

### 7.2. Expanding the Taxonomic and Sample Base

The extent of the current knowledge gap can be illustrated quantitatively. Among the approximately 90 extant cetacean species worldwide, systematic semen cryopreservation studies have been reported for only four species: the bottlenose dolphin (*Tursiops truncatus*), killer whale (*Orcinus orca*), Pacific white-sided dolphin (*Lagenorhynchus obliquidens*), and beluga (*Delphinapterus leucas*). Successful artificial insemination using frozen-thawed spermatozoa has been confirmed in three of these four species. Even basic sperm morphology has been described for only 14 cetacean species across six families. These figures indicate that the current knowledge base in cetacean reproductive science is derived from a small fraction of cetacean diversity less than 5% of all species have been the subject of cryopreservation studies, and successful AI has been achieved in only about 3%. Findings from these few species cannot be assumed to be broadly applicable across the order Cetacea.

The limited availability of reproductively mature males represents one of the greatest barriers to developing evidence-based cryopreservation protocols. Nearly all published studies in this field involve very small sample sizes, often fewer than ten males per species, with consequences for statistical power, assessment of inter-individual variation, and reproducibility of findings. What is often overlooked is that this limitation is not merely a technical nuisance but a fundamental constraint: without adequate sample sizes, even the most sophisticated assessment tools cannot yield generalisable conclusions, and protocol optimisation efforts become inherently underpowered. This is why we identify expanding the taxonomic and sample base as the priority with the highest long-term impact, despite its significant practical constraints including access to rare and protected species, limited numbers of reproductively mature males in both wild and captive populations, and the logistical challenges of sampling in aquatic environments. Progress will require long-term investment in training, field sampling, multi-institutional collaboration, and integration with ongoing conservation programs. Realistically, a stepwise approach is necessary: priority should be given to species with stable captive populations and established handling protocols, with findings gradually extended to more vulnerable species as techniques and collaborations mature.

### 7.3. Developing Functional Sperm Assessment Tools

At present, sperm evaluation in cetaceans such as the Yangtze finless porpoise remains largely limited to conventional parameters of fresh semen, including morphology, concentration, motility, and viability. However, the relationship between post-thaw motility and true fertilizing capacity is not always reliable. For example, studies in boars have shown that post-thaw motility has limited predictive value for IVF success, whereas functional traits such as sperm binding to oviductal epithelial cells more accurately reflect fertilizing competence [66,101]. These findings highlight the need to move beyond conventional semen quality indicators and establish a multi-parameter assessment system for cetacean sperm cryopreservation.

Such a system should distinguish among three levels of evaluation: basic laboratory quality indicators, functional biomarkers, and actual fertility outcomes. Basic indicators, including motility, viability, morphology, and concentration, provide a useful initial screen but may not reliably predict fertilizing capacity. Functional biomarkers, such as plasma membrane integrity, acrosome status, detailed motility characteristics, and DNA fragmentation, provide a more biologically relevant assessment of sperm competence. These can be evaluated using established methods such as PI/FITC-PNA dual staining, computer-assisted sperm analysis (CASA), and sperm chromatin structure assay or sperm chromatin dispersion testing (SCSA/SCD). Nevertheless, functional biomarkers should be interpreted cautiously, because improved laboratory parameters do not necessarily translate into improved reproductive success.

Functional in vitro assays provide a more direct assessment of fertilizing potential than conventional semen parameters. In bottlenose dolphins, heterologous IVF using bovine oocytes has been used to evaluate frozen-thawed spermatozoa and has demonstrated zona penetration and early embryo development [53]. This approach provides an important reference for endangered cetaceans, for which homologous oocytes are rarely available. However, heterologous assays require species-specific validation and depend on access to suitable oocyte sources, which cannot be assumed for all cetacean species.

Emerging omics technologies and artificial intelligence (AI)-based image analysis may further enhance functional sperm assessment in cetaceans. Proteomics could identify sperm proteins associated with membrane stability, capacitation, acrosomal function, mitochondrial activity, and DNA repair capacity. Metabolomics could reveal energy metabolism patterns, oxidative stress status, and osmotic adaptation during freeze–thaw stress. Artificial intelligence-assisted image analysis may improve the objectivity of sperm motility, morphology, and acrosome assessment, particularly when sample numbers are small and rare species are involved. However, these approaches should be regarded as complementary tools rather than replacements for fertility-based validation. Their practical value will depend on whether molecular or image-derived biomarkers can be linked to fertilization, embryo development, pregnancy, or live birth outcomes

Ultimately, the strongest validation of any sperm quality assessment method is its ability to predict fertilization, embryo development, pregnancy, and healthy offspring production in the target species. Yet such validation is particularly difficult in endangered cetaceans, where experimental breeding is often ethically and practically constrained. This creates a circular dilemma: functional assays require fertility data for validation, but fertility data depend on the successful application of the assisted reproductive technologies that these assays are intended to support. Therefore, multi-parameter laboratory assessment and heterologous functional assays are feasible and valuable short-term tools, but their clinical relevance will depend on prospective validation against actual reproductive outcomes whenever such data become available.

### 7.4. Post-Thaw Processing and Functional Intervention Strategies

To maximise the fertilising potential of cryopreserved samples, particularly valuable spermatozoa with low motility but preserved structural integrity, refined post-thaw processing and functional intervention should be prioritised in future research. Before considering novel cryoprotectants, however, it is important to recognise the limitations of currently used agents. Glycerol remains the most widely applied permeating cryoprotectant in cetacean semen cryopreservation, but its use is constrained by cytotoxicity at high concentrations or after prolonged exposure, osmotic injury during addition and removal, oxidative stress during freeze–thaw procedures, and potential destabilisation of the plasma membrane through interaction with the lipid bilayer. These limitations are particularly relevant for endangered cetaceans, for which each sample is highly valuable and poor cryosurvival cannot be offset by large sample numbers. The major cryoprotectants with potential relevance to sperm cryopreservation are summarised in Table 6 [33,56,57,58,63,64,80,102,103,104].

Evidence from other mammalian species suggests that several alternative cryoprotectants may merit systematic evaluation in cetaceans. Ethylene glycol is of particular interest because of its lower molecular weight, rapid permeability, and generally reduced toxicity compared with glycerol. Amide cryoprotectants, such as dimethylformamide and methylformamide, may also reduce osmotic stress and have shown utility in selected domestic species. Non-permeating cryoprotectants such as trehalose could be combined with reduced concentrations of permeating CPAs to limit toxicity while maintaining cryoprotective efficacy. Nevertheless, direct extrapolation from other taxa to cetaceans is not appropriate, given the marked interspecific variability in CPA tolerance even among closely related species. Each candidate CPA therefore requires species-specific dose–response testing, including optimisation of concentration, equilibration time, cooling rate, and removal procedure. This process remains highly dependent on sample availability, as discussed in Section 7.2.

Post-thaw processing provides another important route to improve the functional use of cryopreserved spermatozoa. For immotile but structurally intact spermatozoa, two complementary strategies may be considered. First, polarisation-based birefringence selection can identify spermatozoa with preserved head structure despite the absence of motility. In selected human assisted reproduction studies, this approach has been associated with improved clinical outcomes [105,106,107,108]. Second, chemical activation using motility-inducing agents, such as phosphodiesterase inhibitors including pentoxifylline, can transiently stimulate motility in immotile spermatozoa and facilitate their use for intracytoplasmic sperm injection (ICSI) [109]. Although these approaches are technically promising, their application to cetaceans remains speculative and would require careful validation, particularly because post-activation motility does not necessarily indicate intact DNA, normal acrosomal function, or developmental competence.

Post-thaw sperm selection techniques may also improve the quality of cryopreserved samples by enriching viable spermatozoa and removing damaged cells. Given the association between sperm DNA fragmentation and impaired embryo development, reduced implantation, and increased pregnancy loss [110,111], selection methods that improve DNA integrity are especially relevant. Density gradient centrifugation is one such approach. It can remove dead spermatozoa, cellular debris, and residual cryoprotectants while enriching morphologically and functionally superior spermatozoa. In domestic cats, density gradient selection after cryopreservation restored sperm motility, morphology, and DNA integrity to levels approaching those of fresh samples [112]. Whether similar benefits can be achieved in cetaceans remains unknown, but this method is technically feasible and could be adapted for rare samples if sperm loss during processing is carefully minimised.

Antioxidant intervention represents a further strategy to mitigate freeze–thaw-induced oxidative damage [110,113]. Oxidative stress is a major contributor to cryoinjury, arising from excessive reactive oxygen species (ROS) production during freezing, thawing, and subsequent in vitro handling. ROS can attack polyunsaturated fatty acids in the sperm plasma membrane, causing lipid peroxidation, membrane disruption, DNA fragmentation, and reduced fertilising capacity. Because cetacean sperm membranes may be rich in polyunsaturated fatty acids, antioxidant supplementation could be particularly valuable. However, antioxidant efficacy is concentration-dependent, and excessive supplementation may exert pro-oxidant effects. Therefore, systematic dose–response studies are needed before these compounds can be incorporated into cetacean cryopreservation protocols. Major antioxidants with potential relevance are summarised in Table 7 [114,115,116,117,118,119,120,121,122,123,124].

Overall, post-thaw processing strategies have moderate to high technical feasibility. Methods such as density gradient centrifugation, CASA, flow cytometry, antioxidant supplementation, and selected sperm activation procedures are already established in other mammalian systems and could be transferred to cetaceans with appropriate optimisation. Compared with de novo protocol development or fertility assay validation, the technical barriers are relatively low. However, two important caveats remain. First, most evidence supporting these interventions comes from non-cetacean models, and cetacean spermatozoa may respond differently because of their distinct membrane and biochemical characteristics. Second, improvements in laboratory parameters must ultimately be validated against fertilisation, embryo development, pregnancy, or offspring outcomes before these strategies can be confidently applied in conservation breeding programmes. Thus, although post-thaw processing is among the most technologically ready of the proposed priorities, its ultimate impact will depend on progress in species-specific protocol optimisation, sample availability, and functional fertility assessment.

### 7.5. Concluding Remarks and Prioritization

In summary, the combined application of refined post-thaw processing, functional intervention strategies, validated sperm assessment tools, and species-specific protocol optimisation will be essential for maximising the use of cryopreserved spermatozoa from endangered cetaceans and for advancing the practical application of assisted reproductive technologies.

Based on the hierarchical analysis presented above, we propose the following prioritisation. Priority 1, the development of species-specific optimisation frameworks, is the most immediately actionable because it primarily requires a shift in experimental design, data reporting, and comparative interpretation rather than a major technological breakthrough. However, its full implementation depends on Priority 2, the expansion of taxonomic coverage and sample availability. Priority 2 carries the greatest long-term impact and represents the fundamental rate-limiting step in the field; without sufficient biological material from diverse species, neither protocol optimisation nor assay validation can be pursued rigorously. For this reason, despite its practical difficulty, sustained investment in sample access, genome resource banking, and inter-institutional collaboration is essential.

Priority 3, the development of functional sperm assessment tools validated against actual fertility outcomes, is critical for translating laboratory improvements into meaningful conservation outcomes. However, its progress is constrained by the circular validation dilemma discussed above: functional assays require fertility data for validation, whereas fertility data depend on the successful application of the assisted reproductive technologies that these assays are intended to support. This limitation may be gradually addressed through integration with emerging assisted reproductive technologies and, where possible, opportunistic validation using reproductive outcomes from managed or captive populations. Priority 4, the optimisation of post-thaw processing strategies, should be pursued in parallel rather than treated as a prerequisite for other advances. Its technical readiness is comparatively high, but its ultimate impact will depend on progress in sample availability and functional fertility assessment.

This prioritisation is intended to help direct limited research resources toward the most critical gaps in cetacean reproductive science and to promote a more evidence-based approach to future research planning. Although the framework is necessarily aspirational, progress will require not only technical innovation, but also sustained institutional commitment, cross-disciplinary collaboration, and, most importantly, improved access to biological samples, which remains the overarching constraint across all four priorities.

Finally, semen cryopreservation should be viewed as a complementary conservation tool rather than a stand-alone solution for species recovery. Its practical value will depend on integration with habitat protection, threat reduction, demographic monitoring, genetic management, female reproductive monitoring, and broader in situ and ex situ recovery planning.

## Figures and Tables

**Table 1:** Parameters and methods for male reproductive physiology of Yangtze finless porpoise.

Study Focus	Sample Size (Males/Total)	Individual Source(s)	Geographic Origin	Temporal Coverage	Key Parameters Measured	Reference
Ultrasonography (breeding season)	16 males	Wild, single capture event	Tian-e-Zhou Oxbow, Hubei	April 2008 (cross-sectional)	Testicular volume, echogenicity	[36]
Histology/necropsy	27 males (total dataset)	Stranded/deceased individuals	Multiple sites along middle-lower Yangtze	Years scattered (cumulative)	Testis weight, seminiferous tubule diameter, tunica albuginea thickness	[37]
Longitudinal ultrasonography & serum T	4 males (2 net-pen, 2 captive)	Semi-wild (Tian-e-Zhou) & captive (Baiji Dolphinarium)	Hubei (Tian-e-Zhou & Wuhan)	3.5–5.5 years (quarterly to monthly)	Testicular volume, echogenicity, seasonal dynamics, puberty onset	[38]
Fecal steroid validation (male & female)	1 male/3 total (1M, 2F)	Captive	Baiji Dolphinarium, Wuhan	November 2004–February 2006	Fecal progesterone, estrogen, testosterone (physiological validation)	[41]
Serum hormones & mating season inference	1 male (longitudinal); 41 males (cross-sectional)	Captive (longitudinal) & wild (cross-sectional)	Baiji Dolphinarium & various Yangtze sites	Monthly (captive); spring seasons (wild)	Serum testosterone, estradiol, progesterone	[40]
Fecal testosterone seasonality	1 male	Captive	Baiji Dolphinarium, Wuhan	November 2004–February 2006	Fecal testosterone (breeding vs. non-breeding)	[41]

**Table 2:** Comparison of sperm morphometric parameters among five toothed whale species.

Species	*Neophocaena p. asiaeorientalis* [49]	*Tursiops truncatus* [51]	*Lagenorhynchus obliquidens* [50]	*Orcinus orca* [50]	*Delphinapterus leucas* [50]
Sample size (n)	2 males (5 ejaculates)	1 male	1 male	1 male	1 male (post-mortem)
Collection method	Live, massage	Live, electro-ejaculation	Live, behavioural training	Live, behavioural training	Post-mortem (caudal epididymis)
Body weight (kg)	30–45	~193	60–150	5500	1000
Sperm concentration (×10^6^/mL)	4170	—	620	120	—
Total motility (TM, %)	95	—	90	90	40
Total sperm length (μm)	53.31 ± 4.58	~65	62–68	74–82	68–75
Head length (μm)	3.37 ± 0.14	~4.5	3.5–3.8	3.6–4.0	~3.8
Head width, frontal (μm)	1.90 ± 0.10	~2.0	1.4–1.9	3.3–4.0	4–5
Head shape (frontal)	Oval	Elongated oval	Oval, slightly tapered	Nearly square/paddle	Similar to killer whale
Acrosome:postacrosome ratio	~1:1 (45.8%)	~1:1 (~50%)	~3:2 (~60%)	~3:2 (~60%)	~3:2 (~60%)
Post-acrosomal region	No special structures	14–16 longitudinal ridges	16 longitudinal ridges	No ridges; periodic dense bands (~30 nm)	Similar to killer whale
Midpiece mitochondria	4–5 layers	Two types (A/B electron density)	3–5, randomly arranged	4–5, arranged in layers	Spiral helix (70–80 turns)
Jensen’s ring	Not clearly described	Present	Present, moderate density	Present, dense	Present
Data type/presentation	Mean ± SD	Approx. values	Ranges	Ranges	Approx. values

Notes: 1. Abbreviations: “—“ indicates parameter not reported in the cited source; “approx.” indicates approximate value; values are presented as reported in the original publications. 2. Data sources: All values are derived from single studies as indicated in the column headers; no values represent a synthesis of multiple publications. 3. Acrosome coverage: For *L. obliquidens* and *O. orca*, the original reference [50] reports the ratio as “Acrosome:Postacrosome ≈ 1.5:1”; this has been converted to approximate percentage coverage (~60%). For *D. leucas*, the ratio is described as “similar to killer whale” [50]. 4. Sample quality: The beluga (*D. leucas*) sample was collected post-mortem from an individual with a 22-month history of recurrent subcutaneous nocardiosis, treated with amikacin (31 days BID, 120 days SID). This likely contributed to the poor morphological preservation (40% TM, KR 3.0) and may not represent normal healthy individuals [50]. 5. Measurement conventions: Values are presented as mean ± SD where statistically reported; otherwise as ranges or approximate values as per the original descriptions.

**Table 3:** Comparison of semen collection methods in cetaceans.

Method	Typical Sperm Quality	Animal Welfare	Technical Difficulty	Cost	Endangered Species Applicability	Quantitative Evidence Available
EEJ	Moderate-low, urine/prostatic contamination, poor cryosurvival	High stress, invasive, anaesthesia	Very high	High	Not recommended	Very limited; no within-species comparison with voluntary semen collection
PRT	High, good cryosurvival	Excellent, voluntary, low stress	High (months-years training)	Moderate	Suitable for tractable individuals	More data available from trained odontocetes
Post-mortem	Variable, often lower cryosurvival	N/A	High	Moderate-high	Essential for sudden deaths	Variable; strongly affected by post-mortem interval

**Table 4:** Definitions of principal CASA variables.

Variable	Abbreviation	Definition	Unit
Curvilinear Velocity	VCL	The instantaneous velocity along the sperm’s actual trajectory	μm/s
Straight-Line Velocity	VSL	The velocity of sperm movement along the straight line from start point to end point	μm/s
Average Path Velocity	VAP	The velocity of sperm movement along the average smoothed path	μm/s
Linearity	LIN	Straightness of the curvilinear trajectory (VSL/VCL × 100)	%
Straightness	STR	Straightness of the average path (VSL/VAP × 100)	%
Wobble	WOB	Oscillation index of the average path (VAP/VCL × 100)	%
Amplitude of Lateral Head Displacement	ALH	The amplitude of sperm head oscillation perpendicular to the direction of motion	μm
Beat Cross Frequency	BCF	The frequency with which the sperm tail crosses the average path line	Hz

**Table 5:** Advances in cetacean semen cryopreservation and artificial insemination.

Category	Specific Content	*Tursiops truncatus* [56,57,58,61,64]	*Lagenorhynchus obliquidens* [80]	*Orcinus orca* [33,63]
Semen initial processing and assessment	Centrifugation conditions	1. 5000 rpm × 5 min [56] 2. 250× *g* × 5 min [57] 3. 2000× *g* × 10 min [61] 4. NR [58,64]	NR	NR [33,63]
	Sperm concentration adjustment	1. 4 × 10^8^/mL [56] 2. 400 × 10^8^/mL [57] (a) 3. 200 × 10^6^/mL (final, LP1) [58] 4. NR [61,64]	Not explicitly adjusted	1. Not explicitly adjusted [33] 2. Diluted to 15–25 × 10^6^/mL for CASA [63]
	Fresh semen quality	1. Total 84.4% [64] 2. Total 84.5%, progressive 69.1% [56] 3. Total range 77.5–91.7%, pH 7.8–8.0 [57]	TM 95.3% (M2)/88.0% (M3); PM 93.5%/87.0%; Viability 88.4%/92.2%; SMI 467.5/434.8; pH 8.0–8.1	1. Total motility 92.2 ± 6.3%, progressive 85.4 ± 6.9%, SMI 401.7 ± 61.7, VAP 259.1 ± 53.9 μm/s, VCL 316.2 ± 59.9 μm/s, viability 89.6 ± 9.0%, acrosome intact 89.8 ± 9.2%, normal morphology 90.4 ± 6.8% [63]; 2. Total 90.5%, progressive 94.2% [33]
	DNA fragmentation	SCDt, SFI 0.7–2.0% at T0 [57]	NR	NR (b)
	Salinity/pH effect on motility	Optimal 8–15 ppt, Ph 7.0 [64]; seminal plasma pH 7.42 [61]	NR	NR [33,63]
CPA selection and optimization	Penetrating CPA	Glycerol: 3% [57];6% (pellet, TTF, LP1), 7% (straw), 3% (programmable) [64]; 15% (HSPM) [56]	Glycerol: 6% (EYC); 5% (BF5F)	Glycerol: 3–9% tested, optimal 3–6%; 6% used in DF method [63]; Final 7% [33]
	Non-penetrating CPA	1. Egg yolk (20%), sugars, glutathione, antibiotics [56,57,64] 2. Egg yolk-free: LP1, HSPM [58]	Egg yolk (20%)	1. Egg yolk (20%) in BF5F, Biladyl^®^, EYC [63] 2. Egg yolk (20%), BF5F, Biladyl^®^, EYC [33]
Extender formulation	Base components	1. Reagent I/II [56] 2. Tris-citric acid-fructose-egg yolk [57] 3. EYC, Biladyl^®^, TYB [64] 4. TTF, LP1, HSPM [58]	EYC (Na citrate-egg yolk); BF5F (TES-Tris-fructose-glucose-egg yolk)	1. BF5F (TES-Tris-glucose-fructose-egg yolk); Biladyl^®^ (Tris-citric acid-fructose-egg yolk); EYC (Na citrate-egg yolk) [63] 2. BF5F most effective [63]
	Osmolality/pH	1. 345 ± 5 mOsm/kg [56] 2. 310 mOsm/kg, pH 7.3 [57] 3. Fresh pH 7.8–8.0 [58]	BF5F: 330 ± 5 mOsm/kg, pH 7.0	1. Fresh seminal plasma: 359.1 ± 10.2 mOsm/kg, pH 7.4 ± 0.3 [63] 2. Biladyl^®^ pH 7.0 ± 0.1 [63] 3. BF5F osmolality NR [33]
Freezing protocol	Freezing method	1. LN_2_ vapour [56,57,58,61] 2. Programmable freezer [64] 3. Dry ice pellets [64]	Straw (LN_2_ vapour, 4.5 cm, 10 min); Directional freezing	1. Straw (LN_2_ vapour; SLOW/MED/FAST rates) [63] 2. Directional freezing (DF) [63] 3. Straw LN_2_ vapour [33]
	Cooling rate	1. Programmable: −100 °C/min → −200 °C/min [64] 2. TTF −0.5 °C/min, LP1 −0.27 °C/min, HSPM −0.5 °C/min [58]	Straw: −0.27 °C/min (to 5 °C) + −12 °C/min (vapour); Directional: −0.2 °C/min + 1 mm/s	1. Straw: SLOW—10.7 °C/min, MED—14.6 °C/min, FAST—15.2 °C/min [63] 2. Directional: 1 mm/s through −50 °C zone [63]
	Packaging	1. 0.25 mL straws [57] 2. Cryovials [56] 3. 0.5 mL straws [58,64] 4. 3 mL cryovials (HSPM) [58]	0.5 mL straws; 9 mL hollow tubes (directional)	1. 0.5 mL straws [63]; 2. 2 mL hollow tubes (directional) [63]
Thawing protocol	Thawing conditions	1. 37 °C for 50 s [57,58] 2. 36 °C for 0.5 h [56] 3. 35 °C for 1 min [64]	Straw: 35 °C for 1 min (8.3 °C/s); Directional: air 90 s + 35 °C water bath	1. Straw: 35 °C for 30 s (8.3 °C/s) [63] 2. Directional tube: air 45 s (−100 to −195 °C at 126.6 °C/min) + 35 °C water bath 45 s (−100 to 26 °C at 171.1 °C/min) [63] 3. 1:1 dilution after thawing [33]
Post-thaw assessment	Post-thaw motility	1. TTF total 56.3%, prog 65.0%; LP1 total 68.8%, prog 73.8%; HSPM total 65.0%, prog 75.0% [58] 2. Pellet 25.7%, LN_2_ 56.0%, Prog 61.8% [64]	Straw: TM 50.8%, PM 48.9%, SMI 202.3; Directional: TM 82.5%, PM 79.7%, SMI 366.7	1. Straw (BF5F, 6% glycerol): TM 50.4 ± 10.8%, PM 44.5 ± 12.1%, SMI 176.3 ± 59.7 (0 h); Directional (6% glycerol): TM 75.5 ± 12.7%, PM 56.7 ± 18.2%, VAP 164.7 ± 43.7 μm/s, VCL 222.1 ± 39.5 μm/s (3 h PT) [63] 2. Total 50.3%, progressive 94.0% [33]
	Post-thaw viability	1. 32.40% [56] 2. TTF 43.0%, LP1 60.5%, HSPM 63.0% [58] 3. LN_2_ 70.2%, Prog 68.6% [64]	Straw: 70.2%; Directional: 91.8%	1. Directional (6% glycerol): 57.9 ± 6.4% (viable + intact acrosome, 3 h PT) [63] 2. 90.6% [33]
	Post-thaw acrosome integrity	TTF 57.5%, LP1 71.5%, HSPM 75.5% [58]	NR	Directional: total acrosome intact 93.3 ± 3.1% (6% glycerol, 3 h PT) [63]
	Post-thaw DNA fragmentation	SFI 1.3–1.8% at T0; LP1 increased after 24 h [58]	NR	Directional freezing may reduce DNA fragmentation compared to straws [63] (b)
	Functional assessment	1. Heterologous IVF [57] 2. AI successful [64] 3. Salinity/pH response [61]; 4. DNA dynamics [58]	AI successful, pregnancy rate 50%	1. AI successful, pregnancy rate 38% [33] 2. Directional freezing significantly superior to straw in vitro, expected to improve in vivo fertility [63]
AI outcome	Minimum effective dose (frozen-thawed)	27 × 10^7^ progressive sperm [64]	26.6 × 10^7^ progressive sperm	NR [63]
	AI pregnancy rate	67% (4/6 with frozen-thawed) [64]	50% (5/10 with frozen-thawed); 63% (excluding cervical inseminations)	38% (1/6 with frozen-thawed; 2/3 with liquid stored) [33]

Notes: a. Sperm concentration in [57] is reported as 400 × 10^8^/mL; this value appears unusually high and may contain a typographical error. Readers are advised to refer to the original publication. b. DNA fragmentation in killer whales was assessed in [63] using PI/FITC-PNA staining (not SCDt); directional freezing was reported to reduce DNA fragmentation compared to straws. c. CASA settings (frame rate 60 Hz, minimum contrast 60, minimum cell size 15 pixels, VAP threshold for progressive >20 μm/s, STR > 70%) were standardized within [63] but varied across studies; direct comparisons should be made with caution. d. Centrifugation forces expressed as rpm were not converted to × g due to unreported rotor radii. e. NR = not reported in the cited study.

**Table 6:** Comparison of cryoprotectants for sperm cryopreservation.

Cryoprotectant	Category	Advantages	Limitations	Evidence in Cetaceans
Glycerol [33,56,57,58,63,64,80,102]	Permeating	Effective inhibition of ice crystal formation; widely used; inexpensive	Osmotic stress during addition/removal; cytotoxicity at high concentrations or prolonged exposure; possible membrane destabilisation	Most commonly used in bottlenose dolphin, killer whale, and beluga sperm cryopreservation
Ethylene Glycol [103]	Permeating	Tested as 8% EG + 1% glucose but inferior to DMSO combinations; used with DMSO in oocyte models to reduce CPA concentration	Efficacy varies by species/protocol; no direct comparative data with glycerol on physicochemical or protective properties in the provided literature	No documented evaluation or application
Dimethyl sulfoxide (DMSO) [104]	Permeating	High cell permeability; effective cryoprotective capacity; widely used in other taxa	Toxicity at higher concentrations; post-thaw removal may increase handling and osmotic stress	Very limited; systematic cetacean data are lacking

**Table 7:** Antioxidants evaluated in mammalian sperm cryopreservation.

Antioxidant	Mechanism of Action	Evidence in Mammalian Species	Evidence in Cetaceans
Melatonin [114,115,116,117,118]	Direct free radical scavenger; upregulates endogenous antioxidant enzymes such as SOD, CAT, and GPx; protects membrane lipids from peroxidation	Improves post-thaw motility and membrane integrity in boar, ram, goat, and bovine sperm	Limited; no systematic cetacean studies reported to date
Glutathione [119]	Major cellular antioxidant; reduces lipid peroxidation; protects DNA integrity	Enhances motility, viability, and fertilising capacity in bovine sperm	Very limited or absent systematic cetacean evidence
Resveratrol [120,121,122,123]	Polyphenolic ROS scavenger; modulates endogenous antioxidant pathways; may reduce apoptosis-like changes during cryopreservation	Improves post-thaw sperm quality in human sperm	No systematic cetacean evidence reported to date
Catalase [124]	Enzymatic antioxidant that converts H_2_O_2_ to H_2_O; reduces oxidative damage during freeze–thaw	Effective in equine semen; reduces lipid peroxidation	No systematic cetacean evidence reported to date

## Data Availability

Data availability is not applicable to this article as no new data were created or analyzed in this study.

## References

[1] Carlucci R., Capezzuto F., Cipriano G., D’Onghia G., Fanizza C., Libralato S., Maglietta R., Maiorano P., Sion L., Tursi A. (2020). Assessment of cetacean–fishery interactions in the marine food web of the Gulf of Taranto (Northern Ionian Sea, Central Mediterranean Sea). Rev. Fish Biol. Fish..

[2] Lockyer C. (2002). Ecological Aspects of Reproduction of Marine Mammals. Marine Mammals: Biology and Conservation.

[3] Ralls K., Mesnick S.L. (2019). Cetacean Mating Systems. Encyclopedia of Animal Behavior.

[4] Chivers S.J., Danil K. (2023). Interspecific Comparison of Reproductive Strategies. Sex in Cetaceans.

[5] Carouso-Peck S., Goldstein M.H., Fitch W.T. (2021). The many functions of vocal learning. Philos. Trans. R. Soc. B Biol. Sci..

[6] Kasuya T. (1995). Overview of cetacean life histories: An essay in their evolution. Developments in Marine Biology.

[7] Luo D., Guo Y., Liu Z., Guo L., Wang H., Tang X., Xu Z., Wu Y., Sun X. (2024). Endocrine-Disrupting Chemical Exposure Induces Adverse Effects on the Population Dynamics of the Indo-Pacific Humpback Dolphin. Environ. Sci. Technol..

[8] Luo D., Guo L., Sun X., Xie Q., Wang H., Tang X., Liu Z., Huang N., Zeng C., Wu Y. (2025). Climate change and overfishing combine to drive the population decline of the Indo-Pacific humpback dolphins in the Pearl River Estuary from the Northern South China Sea. Sci. Total Environ..

[9] Minoia L., Consales G., Mazzariol S., Mancusi C., Terracciano G., Ceciarini I., Capanni F., Neri A., D’Agostino A., Marsili L. (2023). Preliminary assessment of persistent organic pollutants (POPs) in tissues of Risso’s dolphin (*Grampus griseus*) specimens stranded along the Italian coasts. Mar. Pollut. Bull..

[10] Scott J.L., Birdsall C., Robinson C.V., Dares L., Dracott K., Jones K., Purdy A., Barrett-Lennard L. (2024). The WhaleReport Alert System: Mitigating threats to whales with citizen science. Biol. Conserv..

[11] Gao A., Zhou K. (1993). Growth and reproduction of three populations of finless porpoise, *Neophocaena phocaenoides*, in Chinese waters. Aquat. Mamm..

[12] Mei Z., Zhang X., Huang S.L., Zhao X., Hao Y., Zhang L., Qian Z., Zheng J., Wang K., Wang D. (2014). The Yangtze finless porpoise: On an accelerating path to extinction?. Biol. Conserv..

[13] Huang J., Mei Z., Chen M., Han Y., Zhang X., Moore J.E., Zhao X., Hao Y., Wang K., Wang D. (2020). Population survey showing hope for population recovery of the critically endangered Yangtze finless porpoise. Biol. Conserv..

[14] Wang R.Y., Chen M.M., Wan X.L., Tang B., Hao Y.J., Mei Z.G., Fan F., Wang K.X., Wang D., Zheng J.S. (2023). Microsatellite genetic diversity evaluation and development prediction of the Yangtze finless porpoise population in the Poyang Lake. Acta Hydrobiol. Sin..

[15] Yanez-Ortiz I., Catalan J., Rodriguez-Gil J.E., Miró J., Yeste M. (2022). Advances in sperm cryopreservation in farm animals: Cattle, horse, pig and sheep. Anim. Reprod. Sci..

[16] Sieme H., Oldenhof H., Wolkers W.F. (2015). Sperm Membrane Behaviour during Cooling and Cryopreservation. Reprod. Domest. Anim..

[17] Larbi A., Li C., Quan G. (2024). An updated review on the application of proteomics to explore sperm cryoinjury mechanisms in livestock animals. Anim. Reprod. Sci..

[18] Caviglia A., Espinoza-Muñoz N., Alvear-Arias J.J., Galizia L., Guastaferri F., Zimmermann R., Sigaut L., Amodeo G., González C., Ozu M. (2024). Membrane tension-dependent conformational change of Isoleucine 106 of loop B diminishes water permeability in FaPIP2;1. Protein Sci..

[19] Ribeiro J.C., Bernardino R.L., Gonçalves A., Barros A., Calamita G., Alves M.G., Oliveira P.F. (2023). Aquaporin-7-mediated glycerol permeability is linked to human sperm motility in asthenozoospermia and during sperm capacitation. Cells.

[20] Chiu P.L., Orjuela J.D., de Groot B.L., Aponte Santamaría C., Walz T. (2024). Structure and dynamics of cholesterol-mediated aquaporin-0 arrays and implications for lipid rafts. eLife.

[21] Jung W.H., Lee S.Y., Lee Y., Ahn D.J. (2025). Freezing-driven ionic charge imbalance leads to pore formation and osmotic injury of lipid membranes. Comput. Biol. Med..

[22] Falabella M., Pizzamiglio C., Tabara L.C., Munro B., Abdel-Hamid M.S., Sonmezler E., Macken W.L., Lu S., Tilokani L., Flannery P.J. (2025). Biallelic PTPMT1 variants disrupt cardiolipin metabolism and lead to a neurodevelopmental syndrome. Brain.

[23] Vieira Neto E., Wang M., Szuminsky A.J., Ferraro L., Koppes E., Wang Y., Van’t Land C., Mohsen A.W., Zanatta G., El-Gharbawy A.H. (2024). Mitochondrial bioenergetics and cardiolipin remodeling abnormalities in mitochondrial trifunctional protein deficiency. JCI Insight.

[24] Loomis P.R., Graham J.K. (2008). Individual male variation in cryosurvival and the response of stallion sperm to customized freezing protocols. Anim. Reprod. Sci..

[25] Spinelli L.G., Randi C.B., Mari R.B., Angrimani D.S.R., Carvalho V.L., Meirelles A.C.O., Vergara-Parente J.E., Guimarães J.P. (2021). Morphological description of the male reproductive tract of the clymene dolphin (*Stenella clymene*, Gray, 1850). Acta Zool..

[26] Orbach D.N., Kelly D.A., Solano M., Brennan P.L.R. (2017). Genital interactions during simulated copulation among marine mammals. Proc. R. Soc. B Biol. Sci..

[27] Katsumata E., Jaroenporn S., Ueda Y., Arai K., Katsumata H., Watanabe G., Taya K. (2017). Circulating gonadotropins and testicular hormones during sexual maturation and annual changes in male bottlenose dolphins (*Tursiops truncatus*). J. Vet. Med. Sci..

[28] Robeck T.R., O’Brien J.K. (2004). Effect of cryopreservation methods and precryopreservation storage on bottlenose dolphin (*Tursiops truncatus*) spermatozoa. Biol. Reprod..

[29] Schroeder J.P. (1990). Breeding bottlenose dolphins in captivity. Bottlenose Dolphin.

[30] van der Horst G., Medger K., Steckler D., Luther I., Bartels P. (2018). Bottlenose dolphin (*Tursiops truncatus*) sperm revisited: Motility, morphology and ultrastructure of fresh sperm of consecutive ejaculates. Anim. Reprod. Sci..

[31] Anderson M.J., Dixson A.F. (2002). Sperm competition: Motility and the midpiece in primates. Nature.

[32] Gu N.H., Zhao W.L., Wang G.S., Sun F. (2019). Comparative analysis of mammalian sperm ultrastructure reveals relationships between sperm morphology, mitochondrial functions and motility. Reprod. Biol. Endocrinol..

[33] Robeck T.R., Steinman K.J., Gearhart S., Reidarson T.R., McBain J.F., Monfort S.L. (2004). Reproductive physiology and development of artificial insemination technology in killer whales (*Orcinus orca*). Biol. Reprod..

[34] Xiao Y., Nabi G., Hao Y., Wang D. (2018). Hormonal Regulation of Testicular Development in the Finless Porpoise *Neophocaena asiaeorientalis sunameri*: Preliminary Evidence from Testicular Histology and Immunohistochemistry. Zool. Stud..

[35] Beirão J., Boulais M., Gallego V., O’Brien J.K., Peixoto S., Robeck T.R., Cabrita E. (2019). Sperm handling in aquatic animals for artificial reproduction. Theriogenology.

[36] Wu H.P. (2010). Ultrasound Monitor on Gonad Development in Male Yangtze Finless Porpoise (*Neophocaena phocaenoides asiaeorientalis*). Master’s Thesis.

[37] Li H.Y. (2010). Histological Study on the Reproductive System of the Yangtze Finless Porpoise (*Neophocaena asiaeorientalis asiaeorientalis*). Master’s Thesis.

[38] Yu X.Y. (2015). Gonadal Development Pattern of Male Yangtze Finless Porpoise (*Neophocaena asiaeorientalis asiaeorientalis*) and Its Response to Photoperiod Variation. Ph.D. Thesis.

[39] Jiang X.F. (1998). Studies on the Development and Histological Characteristics of Testis of Yangtze Finless Porpoise (*Neophocaena phocaenoides asiaeorientalis*). Acta Hydrobiol. Sin..

[40] Chen D.Q., Zhao Q.Z., Liu R.J. (1997). Preliminary study on some hormones of *Neophocaena phocaenoides* in the Yangtze River. Acta Theriol. Sin..

[41] Hao Y.J., Nabi G., Deng X.J., Wang D. (2019). Non-invasive Fecal Steroid Measurements for Monitoring the Reproductive Status of a Critically Endangered Yangtze Finless Porpoises (*Neophocaena asiaeorientalis asiaeorientalis*). Front. Endocrinol..

[42] Florman H.M., Fissore R.A. (2015). Fertilization in mammals. Knobil and Neill’s Physiology of Reproduction.

[43] Avidor-Reiss T. (2018). Rapid evolution of sperm produces diverse centriole structures that reveal the most rudimentary structure needed for function. Cells.

[44] Auger J. (2018). Spermatozoa and sperm structure. Encyclopedia of Reproduction.

[45] Gerton G.L., Vadnais M.L. (2018). Structure of the spermatozoon. Encyclopedia of Reproduction.

[46] Teves M.E., Roldan E.R.S. (2022). Sperm bauplan and function and underlying processes of sperm formation and selection. Physiol. Rev..

[47] Downing Meisner A., Klaus A.V., O’Leary M.A. (2005). Sperm head morphology in 36 species of artiodactylans, perissodactylans, and cetaceans (Mammalia). J. Morphol..

[48] Fuentes-Albero M.D.C., Abril Sánchez S., Ros-Santaella J.L., Pintus E., Luongo C., Ruiz Díaz S., Barros García C., Sánchez Calabuig M.J., García Párraga D., García Vázquez F.A. (2021). Characterization of Bottlenose Dolphin (Tursiops truncatus) Sperm Based on Morphometric Traits. Biology.

[49] Li H.Y., Zhang X.F., Wang D., Chen D.Q. (2009). Ultrastructure of the spermatozoa of the Yangtze finless porpoise (*Neophocaena phocaenoides asiaeorientalis*). Anat. Histol. Embryol..

[50] Miller D.L., Styer E.L., Decker S.J., Robeck T. (2002). Ultrastructure of the spermatozoa from three odontocetes: A killer whale (*Orcinus orca*), a Pacific white-sided dolphin (*Lagenorhynchus obliquidens*) and a beluga (*Delphinapterus leucas*). Anat. Histol. Embryol..

[51] Fleming A.D., Yanagimachi R., Yanagimachi H. (1981). Spermatozoa of the Atlantic bottlenosed dolphin, *Tursiops truncatus*. J. Reprod. Fertil..

[52] Cummins J.M., Woodall P.F. (1985). On mammalian sperm dimensions. J. Reprod. Fertil..

[53] Thuwanut P., Srisuwatanasagul S., Wongbandue G., Tanpradit N., Thongpakdee A., Tongthainan D., Manee-In S., Chatdarong K. (2013). Sperm quality and the morphology of cryopreserved testicular tissues recovered post-mortem from diverse wild species. Cryobiology.

[54] Fernández-Santos M.R., Soler A.J., Ramón M., Ros-Santaella J.L., Martínez-Pastor F., Garde J.J. (2011). Effect of post-mortem time on post-thaw characteristics of Spanish ibex (*Capra pyrenaica*) spermatozoa. Anim. Reprod. Sci..

[55] Keeley T., McGreevy P.D., O’Brien J.K. (2011). Characterization and short-term storage of Tasmanian devil sperm collected post-mortem. Theriogenology.

[56] Yuan S.P., Qi J., Liu B., Wang Y., Tang X., Tan Y. (2023). A Cryopreservation Agent for Bottlenose Dolphin Semen and Method for Cryopreserving Semen. Chinese Patent.

[57] Sánchez-Calabuig M.J., García-Vázquez F.A., Laguna-Barraza R., Barros-García C., García-Parraga D., Rizos D., Gutiérrez Adan A., Pérez-Gutíerrez J.F. (2017). Bottlenose Dolphin (*Tursiops truncatus*) Spermatozoa: Collection, Cryopreservation, and Heterologous In Vitro Fertilization. J. Vis. Exp..

[58] Sanchez-Calabuig M.J., Lopez-Fernandez C., Johnston S.D., Blyde D., Cooper J., Harrison K., de la Fuente J., Gosálvez J. (2015). Effect of cryopreservation on the sperm DNA fragmentation dynamics of the bottlenose dolphin (*Tursiops truncatus*). Reprod. Domest. Anim..

[59] Yamamoto K., Kashiwagi N., Otsuka M., Hori T. (2024). Effects of a simple method for determining the time of insemination and different methods on artificial insemination in common bottlenose dolphins (*Tursiops truncatus*). J. Vet. Med. Sci..

[60] Robeck T.R., Steinman K.J., Montano G.A., Katsumata E., Osborn S., Dalton L., Dunn J.L., Schmitt T., Reidarson T., O’Brien J.K. (2010). Deep intra-uterine artificial inseminations using cryopreserved spermatozoa in beluga (*Delphinapterus leucas*). Theriogenology.

[61] Rich J., Cowart J.R., Montano G., Sánchez-Contreras G.J., Orbach D.N. (2025). Salinity and pH affect common bottlenose dolphin (*Tursiops truncatus*) sperm viability. Anim. Reprod. Sci..

[62] Robeck T.R., Steinman K.J., Greenwell M., Ramirez K., Van Bonn W., Yoshioka M., Katsumata E., Dalton L., Osborn S., O’Brien J.K. (2009). Seasonality, estrous cycle characterization, estrus synchronization, semen cryopreservation, and artificial in-semination in the Pacific white-sided dolphin (Lagenorhynchus obliquidens). Reproduction.

[63] Robeck T.R., Gearhart S.A., Steinman K.J., Katsumata E., Loureiro J.D., O’Brien J.K. (2011). In vitro sperm characterization and development of a sperm cryopreservation method using directional solidification in the killer whale (Orcinus orca). Theri-ogenology.

[64] Robeck T.R., Steinman K.J., Yoshioka M., Jensen E., O’Brien J.K., Katsumata E., Gili C., McBain J.F., Sweeney J., Monfort S.L. (2005). Estrous cycle characterisation and artificial insemination using frozen-thawed spermatozoa in the bottlenose dolphin (*Tursiops truncatus*). Reproduction.

[65] Gao R., Wang W., Wang Z., Fan Y., Zhang L., Sun J., Hong M., Pan M., Wu J., Mei Q. (2024). Hibernating/awakening nanomotors promote highly efficient cryopreservation by limiting ice crystals. Adv. Healthc. Mater..

[66] Farshad A., Wehrend A. (2025). MitoQ as a mitochondria-targeted antioxidant in sperm cryopreservation: An updated review on its mechanisms, efficacy, and future perspectives. Antioxidants.

[67] Xi H., Gao X., Qiu L., Wang Y., Qiu Y., Tao Z., Hu M., Jiang X., Yao Q., Kou L. (2025). Melatonin-loaded nanoparticles protecting human sperm from oxidative stress during cryopreservation. Expert Opin. Drug Deliv..

[68] Fleming S.D., Thomson L.K. (2025). The oxidative stress of human sperm cryopreservation. Antioxidants.

[69] Davoudi S., Raemdonck K., Braeckmans K., Ghysels A. (2023). Capric acid and myristic acid permeability enhancers in curved liposome membranes. J. Chem. Inf. Model..

[70] Pogozheva I.D., Armstrong G.A., Kong L., Hartnagel T.J., Carpino C.A., Gee S.E., Picarello D.M., Rubin A.S., Lee J., Park S. (2022). Comparative molecular dynamics simulation studies of realistic eukaryotic, prokaryotic, and archaeal membranes. J. Chem. Inf. Model..

[71] Harayama T., Antonny B. (2023). Beyond fluidity: The role of lipid unsaturation in membrane function. Cold Spring Harb. Perspect. Biol..

[72] Sakuragi T., Nagata S. (2023). Regulation of phospholipid distribution in the lipid bilayer by flippases and scramblases. Nat. Rev. Mol. Cell Biol..

[73] Huang P., Venskutonytė R., Prasad R.B., Ardalani H., de Maré S.W., Fan X., Li P., Spégel P., Yan N., Gourdon P. (2023). Cryo-EM structure supports a role of AQP7 as a junction protein. Nat. Commun..

[74] Hai E., Li B., Zhang J., Zhang J. (2024). Sperm freezing damage: The role of regulated cell death. Cell Death Discov..

[75] Álvarez-Merz I., Fomitcheva I.V., Sword J., Hernández-Guijo J.M., Solís J.M., Kirov S.A. (2022). Novel mechanism of hypoxic neuronal injury mediated by non-excitatory amino acids and astroglial swelling. Glia.

[76] Kerst S., Hoogterp L., Breur M., van Rooijen-van Leeuwen G.M., Bugiani M., Sah R., Mansvelder H.D., van der Knaap M.S., Min R. (2026). Astrocyte-specific deletion of LRRC8A causes neurological dysfunction but not chronic white matter edema. Neurobiol. Dis..

[77] Cao L., Wang L., Li Z., Wei X., Ding J., Zhou C., Chen X., Huang Z., Shao Z., Shen J. (2025). Radiotherapy enhances anticancer CD8 T cell responses by cGAMP transfer through LRRC8A/C volume-regulated anion channels. Sci. Immunol..

[78] Yuen Q.W., Brook F.M., Kinoshita R.E., Ying M.T. (2009). Semen collection and ejaculate characteristics in the Indo-Pacific bottlenose dolphin (Tursiops aduncus). J. Androl..

[79] Serafini S., O’Flaherty C. (2025). Novel insights into the lipid signalling in human spermatozoa. Hum. Reprod..

[80] Mhimdi M., Selmi S., Taamalli W., Sut S., Sebai H., Dall’acqua S. (2026). Protective effects of *Myrtus communis* essential oil against bisphenol A-induced sperm dysfunction: Insights from lipidomic, amino acid profiling, oxidative stress and molecular docking. Antioxidants.

[81] Zhu Z., Li W., Yang Q., Zhao H., Zhang W., Adetunji A.O., Hoque S.A.M., Kou X., Min L. (2024). Pyrroloquinoline quinone improves ram sperm quality through its antioxidative ability during storage at 4 °C. Antioxidants.

[82] Su G., Liu Z., Xue H., Zhao X., Yang L., Wu D., Hai C., Liu X., Song L., Bai C. (2025). Spirulina polysaccharides improve postthaw sperm quality in bulls by inhibiting the activation of pathways related to protein kinase A. Int. J. Biol. Macromol..

[83] Xiao S., Riordon J., Lagunov A., Ghaffarzadeh M., Hannam T., Nosrati R., Sinton D. (2023). Human sperm cooperate to transit highly viscous regions on the competitive pathway to fertilization. Commun. Biol..

[84] Belardin L.B., Antoniassi M.P., Camargo M., Intasqui P., Bertolla R.P. (2023). Separating the chaff from the wheat: Antibody-based removal of DNA-fragmented sperm. Hum. Reprod..

[85] Kato Y., Matsuda Y., Uto T., Tanaka D., Ishibashi K., Ishizaki T., Ohta A., Kobayashi A., Hazawa M., Wong R.W. (2023). Cell-compatible isotonic freezing media enabled by thermo-responsive osmolyte-adsorption/exclusion polymer matrices. Commun. Chem..

[86] Castro-Arnau J., Chauvigné F., Toft-Bertelsen T.L., Finn R.N., MacAulay N., Cerdà J. (2024). Aqp4a and Trpv4 mediate regulatory cell volume increase for swimming maintenance of marine fish spermatozoa. Cell. Mol. Life Sci..

[87] Ribeiro J.C., Bernardino R.L., Jorge P., Geraldo M., Vieira E., Santos R., Barros A., Touré A., Castro T.G., Cavaco-Paulo A. (2025). A novel CFTR-AQP7 protein complex regulates glycerol transport and motility of human sperm. Hum. Reprod..

[88] Tseng H.Y., Chen C.J., Wu Z.L., Ye Y.M., Huang G.Z. (2022). The non-contact-based determination of the membrane permeability to water and dimethyl sulfoxide of cells virtually trapped in a self-induced micro-vortex. Lab Chip.

[89] Asadi E., Najafi A., Benson J.D. (2022). Exogenous melatonin ameliorates the negative effect of osmotic stress in human and bovine ovarian stromal cells. Antioxidants.

[90] Dan N., Shelake S., Luo W.C., Rahman M., Lu J., Bogner R.H., Lu X. (2024). Impact of controlled ice nucleation on intracellular dehydration, ice formation and their implications on T cell freeze-thaw viability. Int. J. Pharm..

[91] Kuželová L., Svoradová A., Baláži A., Vašíček J., Langraf V., Kolesárová A., Sláma P., Chrenek P. (2024). Enhancing of rabbit sperm cryopreservation with antioxidants Mito-Tempo and berberine. Antioxidants.

[92] Sui H., Wang X., Hu K., Zuo X., Li H., Diao Z., Feng J., Zhang Y., Cao Z. (2025). Effects of mogroside V on quality and antioxidant activity of boar frozen-thawed sperm. Antioxidants.

[93] Bu Y., Shi D., Li J., Jiang X., Chen Y., Wu Z., Li W., Li L., Zhang S., Wei H. (2025). Methyl gallate enhances post-thaw boar sperm quality by alleviating oxidative stress and preserving mitochondrial function. Antioxidants.

[94] Lin M., Cao H., Li J. (2023). Control strategies of ice nucleation, growth, and recrystallization for cryopreservation. Acta Biomater..

[95] Wu X., Yao F., Zhang H., Li J. (2021). Antifreeze proteins and their biomimetics for cell cryopreservation: Mechanism, function and application—A review. Int. J. Biol. Macromol..

[96] Wu X., Yi X., Zhao B., Zhi Y., Xu Z., Cao Y., Cao X., Pang J., Yung K.K.L., Zhang S. (2023). The volume regulated anion channel VRAC regulates NLRP3 inflammasome by modulating itaconate efflux and mitochondria function. Pharmacol. Res..

[97] Zhao T.J., Liu L., Chang Y.Q., Zhan Y.Y. (2020). Research progress on cryopreservation of stem cells in spermatogonia and embryos in animals in aquaculture: A review. J. Dalian Ocean Univ..

[98] Kawasaki T., Siegfried K.R., Sakai N. (2016). Differentiation of zebrafish spermatogonial stem cells to functional sperm in culture. Development.

[99] Lacerda S.M.S.N., Batlouni S.R., Costa G.M.J., Segatelli T.M., Quirino B.R., Queiroz B.M., Kalapothakis E., França L.R. (2010). A new and fast technique to generate offspring after germ cells transplantation in adult fish: The Nile tilapia (*Oreochromis niloticus*) model. PLoS ONE.

[100] Gao F., He Q.F., Wu S.H., Wang S.W., Xu X.R., Kang J., Zhang Y., Quan F.S. (2022). Mammalian gametes cryopreserved and applied to technical strategies for the protection of rare and endangered animals. Chin. J. Anim. Vet. Sci..

[101] Daigneault B.W., McNamara K.A., Purdy P.H., Krisher R.L., Knox R.V., Miller D.J. (2014). Novel and traditional traits of frozen-thawed porcine sperm related to in vitro fertilization success. Theriogenology.

[102] Bryant S.J., Awad M.N., Elbourne A., Christofferson A.J., Martin A.V., Meftahi N., Drummond C.J., Greaves T.L., Bryant G. (2022). Deep eutectic solvents as cryoprotective agents for mammalian cells. J. Mater. Chem. B.

[103] Hossen S., Sukhan Z.P., Cho Y., Lee W.K., Kho K.H. (2022). Antioxidant activity and oxidative stress-oriented apoptosis pathway in saccharides supplemented cryopreserved sperm of Pacific abalone, *Haliotis discus hannai*. Antioxidants.

[104] Modaresi S., Pacelli S., Chakraborty A., Coyle A., Luo W., Singh I., Paul A. (2024). Engineering a microfluidic platform to cryopreserve stem cells: A DMSO-free sustainable approach. Adv. Healthc. Mater..

[105] Baccetti B. (2004). Microscopical advances in assisted reproduction. J. Submicrosc. Cytol. Pathol..

[106] Gianaroli L., Magli M.C., Collodel G., Moretti E., Ferraretti A.P., Baccetti B. (2008). Sperm head‘s birefringence: A new criterion for sperm selection. Fertil. Steril..

[107] Ribeiro M.A., Broi M.G.D., Rose M.B., Garolla A., Foresta C., Bragheto A.M.D.S., Hardy D.G.F. (2023). Sperm selection by birefringence: A promising non-invasive tool to improve ICSI outcomes. JBRA Assist. Reprod..

[108] Magli M.C., Crippa A., Perruzza D., Azzena S., Graziosi S., Coppola F., Tabanelli C., Ferraretti A.P., Gianaroli L. (2023). Birefringence properties of human immotile spermatozoa and ICSI outcome. Reprod. Biomed. Online.

[109] Mahaldashtian M., Khalili M.A., Nottola S.A., Woodward B., Macchiarelli G., Miglietta S. (2021). Does in vitro application of pentoxifylline have beneficial effects in assisted male reproduction?. Andrologia.

[110] Borges E., Zanetti B.F., Setti A.S., Braga D.P.A.F., Provenza R.R., Iaconelli A. (2019). Sperm DNA fragmentation is correlated with poor embryo development, lower implantation rate, and higher miscarriage rate in reproductive cycles of non-male factor infertility. Fertil. Steril..

[111] Sivanarayana T., Ravi Krishna C., Jaya Prakash G., Krishna K.M., Madan K., Sudhakar G., Rama Raju G.A. (2013). Sperm DNA fragmentation assay by sperm chromatin dispersion (SCD): Correlation between DNA fragmentation and outcome of intracytoplasmic sperm injection. Reprod. Med. Biol..

[112] Munoz-Maceda A., Priego-Gonzalez A., Barroso-Arévalo S., Blázquez J.C., Fernandez-Gonzalez R., Garcia-Vazquez F.A., Gutiérrez-Adán A., Roldan E.R.S., Sánchez-Calabuig M.J. (2026). Impact of different cryopreservation protocols on epididymal sperm quality and subsequent in vitro embryo production in domestic cats. Theriogenology.

[113] Amidi F., Pazhohan A., Shabani Nashtaei M., Khodarahmian M., Nekoonam S. (2016). The role of antioxidants in sperm freezing: A review. Cell Tissue Bank.

[114] Li X., Xu D., Xu H., Cao P. (2026). Melatonin supplementation in sex-sorted Nili-Ravi buffalo semen: Effect on sperm quality, subsequent in vitro embryo development, and pregnancy outcomes. Antioxidants.

[115] Cardenas-Padilla A.J., Jimenez-Trejo F., Cerbon M., Medrano A. (2024). The role of melatonin on caprine (*Capra hircus*) sperm freezability: A review. Antioxidants.

[116] Gallo A., Esposito M.C., Tosti E., Boni R. (2021). Sperm motility, oxidative status, and mitochondrial activity: Exploring correlation in different species. Antioxidants.

[117] Li J.H., Liu J.L., Li J.H., Zhou A.D., Wang H., Qiu F., Xie X.L., Wang Q. (2026). Melatonin attenuates reproductive toxicity of prothioconazole and its metabolite via spermatogenesis and oxidative stress. Commun. Biol..

[118] Benitez Mora M.P., Del Prete C., Longobardi V., De Canditiis C., Natale A., Cocchia N., Esposito R., Piscopo F., Sicari A., Vinale F. (2024). Incubating frozen-thawed buffalo sperm with olive fruit extracts counteracts thawing-induced oxidative stress and improves semen quality. Theriogenology.

[119] Njoroge W.E., Zhu Z., Umehara T., Yamanaka T., Zeng W., Okazaki T., Shimada M. (2025). Synthesis of functional enzymes involved in glutathione production during linear motility in boar sperm. Free Radic. Biol. Med..

[120] Tachibana R., Takeuchi H., Yoshikawa-Terada K., Maezawa T., Nishioka M., Takayama E., Tanaka H., Tanaka K., Hyon S.H., Gen Y. (2023). Carboxylated poly-L-lysine potentially reduces human sperm DNA fragmentation after freeze-thawing, and its function is enhanced by low-dose resveratrol. Cells.

[121] Bang S., Qamar A.Y., Tanga B.M., Fang X., Cho J. (2021). Resveratrol supplementation into extender protects against cryodamage in dog post-thaw sperm. J. Vet. Med. Sci..

[122] Hussain T., Fayyaz M.H., Hameed A., Hassan Andrabi S.M., Kausar R., Mubashir Y., Batool I., Shahzad M., Omur A.D. (2025). Effect of resveratrol on post-thaw motility, kinematics, structural parameters and antioxidant/oxidant status of Kamori buck spermatozoa. Cryobiology.

[123] Assunção C.M., Mendes V.R.A., Brandão F.Z., Batista R.I.T.P., Souza E.D., Carvalho B.C., Quintão C.C.R., Raposo N.R.B., Camargo L.S.A. (2021). Effects of resveratrol in bull semen extender on post-thaw sperm quality and capacity for fertilization and embryo development. Anim. Reprod. Sci..

[124] Medica A.J., Swegen A., Seifi-Jamadi A., McIntosh K., Gibb Z. (2025). Catalase in unexpected places: Revisiting H_2_O_2_ detoxification pathways in stallion spermatozoa. Antioxidants.

