# Correction: Wang et al. Current Advances in Cetacean Semen Cryopreservation and Their Application to Yangtze Finless Porpoise Conservation. *Animals* 2026, *16*, 2191

**DOI:** 10.3390/ani16182967

**Published:** 2026-09-21

**Authors:** Qingyue Wang, Congping Ying, Chu Wang, Jialu Zhang, Danqing Lin, Kai Liu, Shengyan Su

**Affiliations:** 1 Key Laboratory of Freshwater Fisheries and Germplasm Resources Utilization, Ministry of Agriculture and Rural Affairs, Freshwater Fisheries Research Center, Chinese Academy of Fishery Sciences, Wuxi 214081, China; 15094386833@163.com (Q.W.); yingcongping@ffrc.cn (C.Y.); 19281843551@163.com (C.W.); zhangjialu@ffrc.cn (J.Z.); lindq@ffrc.cn (D.L.); 2 Wuxi Fisheries College, Nanjing Agricultural University, Wuxi 214081, China

## Missing Citation

In the original publication [1], the citation Beirão, J.; Boulais, M.; Gallego, V.; O’Brien, J.K.; Peixoto, S.; Robeck, T.R.; Cabrita, E. Sperm handling in aquatic animals for artificial reproduction. *Theriogenology* **2019**, *133*, 161–178. https://doi.org/10.1016/j.theriogenology.2019.05.004 was inadvertently omitted from the legend of Figure 2. The citation has now been inserted into the legend of Figure 2 in 3. Overview of Cetacean Reproductive Physiology and Semen Characteristics, 3.1. Anatomical and Physiological Features of the Male Cetacean Reproductive System, and reads:

Figure 2. External genitalia of a male bottlenose dolphin (*T. truncatus*) [35] (A) and a male Yangtze finless porpoise (*N. asiaeorientalis asiaeorientalis*) (B). The anatomical features shown provide the basis for semen collection and the development of species-appropriate cryopreservation procedures.

In addition, text in 2. Literature Search Strategy and Review Methodology, 2.3. Literature Screening Process, Paragraph 3—“Therefore, this review cites a total of 123 references”—is corrected to the following:

“Therefore, this review cites a total of 124 references.”

With this correction, the order of some references has been adjusted accordingly. The authors state that the scientific conclusions are unaffected. This correction was approved by the Academic Editor. The original publication has also been updated.
